# Gene expression profiling reveals a conserved microglia signature in larval zebrafish

**DOI:** 10.1002/glia.23717

**Published:** 2019-09-11

**Authors:** Julie Mazzolini, Sigrid Le Clerc, Gregoire Morisse, Cédric Coulonges, Laura E. Kuil, Tjakko J. van Ham, Jean‐François Zagury, Dirk Sieger

**Affiliations:** ^1^ Centre for Discovery Brain Sciences University of Edinburgh Edinburgh UK; ^2^ Laboratoire GBCM, EA7528, Conservatoire National des Arts et Métiers HESAM Université Paris France; ^3^ Department of Clinical Genetics, Erasmus MC University Medical Center Rotterdam Rotterdam The Netherlands

**Keywords:** brain, evolution, microglia, RNA sequencing, transcriptome, zebrafish

## Abstract

Microglia are the resident macrophages of the brain. Over the past decade, our understanding of the function of these cells has significantly improved. Microglia do not only play important roles in the healthy brain but are involved in almost every brain pathology. Gene expression profiling allowed to distinguish microglia from other macrophages and revealed that the full microglia signature can only be observed in vivo. Thus, animal models are irreplaceable to understand the function of these cells. One of the popular models to study microglia is the zebrafish larva. Due to their optical transparency and genetic accessibility, zebrafish larvae have been employed to understand a variety of microglia functions in the living brain. Here, we performed RNA sequencing of larval zebrafish microglia at different developmental time points: 3, 5, and 7 days post fertilization (dpf). Our analysis reveals that larval zebrafish microglia rapidly acquire the core microglia signature and many typical microglia genes are expressed from 3 dpf onwards. The majority of changes in gene expression happened between 3 and 5 dpf, suggesting that differentiation mainly takes place during these days. Furthermore, we compared the larval microglia transcriptome to published data sets of adult zebrafish microglia, mouse microglia, and human microglia. Larval microglia shared a significant number of expressed genes with their adult counterparts in zebrafish as well as with mouse and human microglia. In conclusion, our results show that larval zebrafish microglia mature rapidly and express the core microglia gene signature that seems to be conserved across species.

## Main points

- Larval zebrafish microglia express the core microglia signature.
- 3097 genes show differential expression during development in larval zebrafish microglia.
- Larval zebrafish microglia show significant similarities to adult zebrafish microglia, mouse microglia and human microglia.

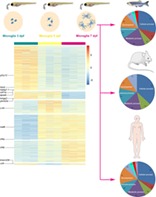

## 1 INTRODUCTION

Microglia represent the tissue‐resident macrophage population of the brain. Microglia are derived from primitive macrophages that colonize the central nervous system (CNS) early during development where they differentiate into mature microglia (Prinz, Erny, & Hagemeyer, 2017). This differentiation is reflected in changes in their gene expression as well as the appearance of the typical ramified morphology of microglia. Microglia caught significant attention over the past decade as these cells do not only have crucial functions during physiology but are involved in almost every type of CNS pathology (Salter & Stevens, 2017; Song & Colonna, 2018). Advances in sequencing technology have provided an in‐depth understanding of the microglia gene expression signature. This not only allows a clear discrimination of microglia from other brain cells, but also other populations of macrophages (Butovsky et al., 2013). In line with this, based on RNA sequencing of mouse microglia in various studies, a microglia core gene signature could be determined. Among other genes, this core signature includes the genes *Cx3cr1*, *Hexb*, *Itgb5*, *Olfml3*, *P2ry12*, *P2ry13*, *Rnase4*, *Slc2a5*, *Tmem119*, *Trem2*, *Gpr34*, *Siglech*, *Gpr84*, and *Socs3* (Beutner et al., 2013; Butovsky et al., 2013; Chiu et al., 2013; Gautier et al., 2012; Hickman et al., 2013; Zhang et al., 2014).

The zebrafish (*Danio rerio*) has become a very popular model for biomedical studies. The optical transparency of the zebrafish larva, combined with the ease of genetic and pharmacological manipulation, make it an ideal model for in vivo imaging studies. Based on these advantages, several elegant studies have addressed microglial functions using the zebrafish as a model (Casano, Albert, & Peri, 2016; Herbomel, Thisse, & Thisse, 2001; Oosterhof et al., 2016; Peri & Nüsslein‐Volhard, 2008; Rossi, Casano, Henke, Richter, & Peri, 2015; Shen, Sidik, & Talbot, 2016; Shiau, Monk, Joo, & Talbot, 2013; Sieger, Moritz, Ziegenhals, Prykhozhij, & Peri, 2012; Svahn et al., 2013; Xu, Wang, Wu, Jin, & Wen, 2016). In zebrafish, a subpopulation of primitive macrophages from the yolk sac colonize the brain early during development from 48 hr post fertilization (hpf) onwards. Once in the brain, these macrophages then rapidly differentiate into early microglia over the next 24 hr (Herbomel et al., 2001). This differentiation has been described based on the down‐regulation of *l‐plastin*, the strong up‐regulation of *apoE* and the appearance of a ramified morphology (Herbomel et al., 2001). Furthermore, typical microglia marker genes like *p2ry12* are expressed in zebrafish microglia immediately upon brain colonization at 3 dpf (Sieger et al., 2012). Several studies revealed that early larval zebrafish microglia, at 3 and 4 dpf, are fully functional. These cells have been shown to clear apoptotic neurons, to directly interact with highly active neurons and to respond to neuronal injuries (Li, Du, Liu, Wen, & Du, 2012; Mazaheri et al., 2014; Peri & Nüsslein‐Volhard, 2008; Sieger et al., 2012). Importantly, the mechanisms underlying these functions are the same as those employed by mammalian microglia, suggesting a high degree of conservation across species (Sieger & Peri, 2013). Interestingly, in zebrafish, these larval microglia seem to be replaced by a second wave of definitive microglia that persist throughout adulthood and are derived from cmyb‐dependent hematopoietic stem cells (Ferrero et al., 2018; Xu et al., 2015). The transcriptome of these adult zebrafish microglia was recently analyzed and a comparison with available data sets from mouse showed that a large fraction of the mouse microglia specific gene expression signature is conserved in the zebrafish (Oosterhof et al., 2016).

To date, many studies have used larval zebrafish to study microglia because of the optical transparency, which allows high‐resolution in vivo imaging. However, the gene expression profile of larval zebrafish microglia has not been addressed so far. Thus, to gain an in‐depth understanding of the gene expression profile of microglia during larval development we performed RNA sequencing of microglia at different time points during zebrafish development. Microglia were isolated at 3, 5, and 7 dpf and their gene expression profiles were analyzed and compared to available data sets from adult zebrafish microglia, embryonic, and adult mouse microglia, as well as human microglia. Our results show that zebrafish microglia undergo a rapid differentiation which is reflected in the strong upregulation of many microglia specific genes at 3 dpf. Furthermore, our data show that larval zebrafish microglia share many genes with adult zebrafish microglia as well as with mouse and human microglia. In conclusion, our new gene expression data combined with previous functional studies on larval microglia, underscore the suitability of the larval zebrafish to study microglial functions and mechanisms.

## 2 METHODS

### 2.1 Zebrafish maintenance

Animal experimentation was approved by the ethical review committee of the University of Edinburgh and the Home Office, in accordance with the Animal (Scientific Procedures) Act 1986. Zebrafish were housed in a purpose‐built zebrafish facility, in the Queen's Medical Research Institute, maintained by the University of Edinburgh Biological Resources. All zebrafish larvae were kept at 28°C on a 14 hr light/10 hr dark photoperiod. Embryos were obtained by natural spawning from adult *Et(Zic4:Gal4TA4*,*UAS:mCherry)* ^*hmz5*^ referred to as zic4:mCherry (Distel, Wullimann, & Köster, 2009), Tg(XIa.Tubb:dsRED) referred to as NBT:dsRED (Peri & Nüsslein‐Volhard, 2008), Tg(mpeg1:EGFP) (Ellett, Pase, Hayman, Andrianopoulos, & Lieschke, 2011), and wild‐type (WIK), zebrafish strains. Embryos were raised at 28.5°C in embryo medium (E3) and treated with 200 μM 1‐phenyl 2‐thiourea (PTU) (Sigma) from the end of the first day of development for the duration of the experiment to prevent pigmentation.

### 2.2 Mounting, immunohistochemistry, and image acquisition

Whole‐mount immunostaining of samples was performed as previously described (Astell & Sieger, 2017). Briefly, larvae were fixed in 4% PFA/1% DMSO in PBS at room temperature for 2 hr, then washed in PBStx (0.2% Triton X‐100 in 0.01 M PBS) and blocked in 1% goat serum blocking buffer (1% normal goat serum, 1% DMSO, 1% BSA, and 0.7% Triton X‐100 in 0.01 M PBS) for 2 hr prior to incubation with the mouse anti‐4C4 primary antibody (1:50) overnight at 4°C. Samples were washed in PBStx before their incubation with conjugated secondary antibodies (goat anti‐mouse Alexa Fluor 647 [1:200]) (Life Technologies) overnight at 4°C. The samples were washed several times with PBStx and stored in 70% glycerol at 4°C until final mounting in 1.5% low melting point agarose (Life Technologies) in E3 for image acquisition.

Whole‐brain immunofluorescent images were acquired using confocal laser scanning microscopy (Zeiss LSM780; 20×/0.8 objective; 2.30 mm intervals; 633 nm laser line).

Isolated cells from fluorescence‐activated cell sorting (FACS) were centrifuged on glass coverslip pretreated with 0.01% Poly‐l‐lysine (Sigma Aldrich) in ΔH_2_O at 400*g* for 5 min. Cells were fixed with 4% PFA in PBS for 15 min at room temperature, washed twice in PBS then mounted on microscopic slides in 10 μl of Fluoromount‐G® (SouthernBiotech). Samples were acquired using confocal laser scanning microscopy (Zeiss LSM880; 63×/1.4 objective; 50 μm intervals; 633, 561, and 514 nm laser lines).

### 2.3 Image analysis

Analysis of all images was performed in 3D using Imaris (Bitplane, Zurich, Switzerland). To assess microglia morphology, we used the surface‐rendering tool in Imaris 8.0.2, which allowed segmentation of individual cells in 3D. To visualize microglia labeling (4C4/Alx647), NBT:DsRed, and zic4:mCherry signals from isolated cells, we used the section view which allowed viewing of those signals along three coordinate axes (*X*, *Y*, and Z). The surface‐rendering tool was used to build a 3D reconstitution based on the expression of the different signals (Figure S2).

### 2.4 Microglia and macrophage isolation

Microglia were isolated by FACS from heads of 3, 5, and 7 dpf *Et(Zic4:Gal4TA4*,*UAS:mCherry)* ^*hmz5*^ larvae as previously described (Mazzolini, Chia, & Sieger, 2018) whereas macrophages were isolated from whole 28 hpf Tg(mpeg1:EGFP) larvae. FACS allowed cell separation from debris in function of their size (FSC‐A) and granularity (SSC‐A). Single cells were then separated from doublets or cell agglomerates (FSC Singlet; SSC Singlet). From the single‐cell population, a gate was drawn to separate live cells (DAPI−) from dead cells (DAPI+). Unstained and cells incubated with secondary antibody Alx647 only were used as controls to draw gates corresponding to macrophage and microglia populations. Finally, microglia (Alx647+; Figure S1) and macrophages (eGFP+; Figure S4) were segregated from the live cell population gates. FACS data were analyzed and median of fluorescence intensity of microglia staining measured using FlowJo Software (Treestar, Ashland, OR).

To assess sample purity, (Alx647+; DsRed+) and (Alx647+; mCherry+) cells were analyzed then isolated from 7 dpf *Et(Zic4:Gal4TA4*,*UAS:mCherry)* ^*hmz5*^ and Tg(XIa.Tubb:dsRED) larvae labeled for microglia as described above. Isolated cells were then analyzed by microscopy (Figure S2).

### 2.5 RNA extraction and cDNA amplification

All experiments were performed in three replicates with a total number of 600 larvae per replicate. Total RNA extraction from microglial cells was performed using the Qiagen RNeasy Plus Micro kit according to the manufacturer's instructions (Qiagen). RNA sample quality and concentration were determined using the Agilent RNA 6000 Pico kit and an Agilent 2100 Bioanalyser System (Agilent Technologies). For sequencing, all RNA samples with a RIN score >7 were transcribed into cDNA using the Ovation RNA‐Seq System V2 kit according to the manufacturer's instructions (NuGEN). Samples were then sent to Edinburgh Genomics for library synthesis and sequencing. For qPCR, RNA sample quality and concentration were assessed using the LabChip GX Touch Nucleic Acid Analyzer and RNA Pico Sensitivity Assay. All RNA samples with a RIN score >7 were transcribed from the same amount of RNA into cDNA using the SuperScript® III First‐Strand Synthesis System (Invitrogen).

### 2.6 Library synthesis

Sequencing libraries were prepared using the Illumina TruSeq DNA Nano library preparation kit according to manufacturer's instructions with amended shearing conditions (duty factor 10%, PIP 175, cycles/burst 200, duration 40 s) using a 500 ng input of amplified cDNA (Illumina, Inc.). The size selection for the sheared cDNA was set for 350 bp products. Libraries were normalized and ran on 2 HiSeq 4000 lanes with 75‐base paired‐end reads resulting in an average read depth of around 20 million read pairs per sample.

### 2.7 Bioinformatics

The quality control of the sequences was done with FastQC (Andrews, 2010), and Trimmomatic was applied to trim low‐quality reads and adapters (Bolger, Lohse, & Usadel, 2014). We aligned the RNA‐seq reads to the zebrafish reference genome (Ensembl, GRCz11) using STAR v2.6 (Dobin et al., 2012) and transcript were assembled and counted with HTSeq (Anders, Pyl, & Huber, 2015) using annotation from Ensembl (Danio_rerio.GRCz11.93.gtf).

Counts normalization, transformation (*rlog*), and differential expression analysis were performed using DESeq2 (Love, Huber, & Anders, 2014). Normalized data were inspected using Principal Component Analysis (PCA) (Figure 2), and inter‐sample correlation plots (Figure S1). Two types of differential expression analyses were performed using DESeq2: (i) microglia at 3 dpf versus 5 dpf, microglia at 5 dpf versus 7 dpf, microglia at 3 dpf versus 7 dpf; (ii) microglia at 3 or 5 dpf or 7 dpf versus brain cells (from Oosterhof et al., 2016). Differentially expressed genes (DEs) were selected using the following filter criteria: FDR ≤ 0.05 and Fold Change ≥ |2|. Enrichment for gene ontology (GO) terms for individual comparisons was performed using Gorilla (Eden, Navon, Steinfeld, Lipson, & Yakhini, 2009).

The zebrafish microglia expression (i) and (ii) profile were compared with reported microglia expression profiles (Galatro et al., 2017; Matcovitch‐Natan et al., 2016; Oosterhof et al., 2016; Zhang et al., 2014). The analysis was performed in two steps: (a) Pearson correlation of gene expression and (b) gene overlap list enrichment between zebrafish 3, 5, and 7 dpf samples and samples from the other studies. The ZFIN database was used to annotate the genes and to identify zebrafish orthologs (https://zfin.org/). For the correlation analysis, RNA‐seq data were downloaded from GEO (GEO id: GSE86921, GSE52564, GSE79812, and GSE99074). The RNA‐seq were treated according to our protocol described above. Because normalization rescales samples relative to one another, the data were re‐normalized separately for each analysis. The mean expression was computed for biological replicates and we performed a Pearson correlation and the differences of correlation coefficient were tested using Hotelling–Williams test. The significant genes higher expressed in microglia in the different studies were selected according to our criteria (Fold Change ≥2, FDR < 0.05) if possible. Otherwise, the selection criteria are available in Table S12. The gene symbol list from (ii) was intersected with the gene symbol list of genes expressed in adult zebrafish microglia (Oosterhof et al., 2016), adult mouse microglia (Zhang et al., 2014) and adult human microglia (Galatro et al., 2017). We compared the evolution of the developmental microglia gene expression profile by intersecting the gene symbol list from (i) with genes expressed during mouse microglia development (Matcovitch‐Natan et al., 2016).The overlap significance between microglia specific genes from 3, 5, and 7 dpf with other species was calculated using a hypergeometric distribution.

### 2.8 Quantitative PCR

Quantitative (qPCR) amplifications were performed in duplicates in a 20 μl reaction volume containing SsoAdvanced Universal SYBR Green Supermix (Bio‐Rad) using a LightCycler 96 Real‐Time PCR System (Roche). The PCR protocol used was initial denaturation step of 5 min at 95°C, and 45 cycles of 10 s at 95°C, 20 s at 56°C, and 20 s at 72°C. Primers used were:

*Beta‐actin* forward 5′‐CACTGAGGCTCCCCTGAATCCC‐3′.

*Beta‐actin* reverse 5′‐CGTACAGAGAGAGCACAGCCTGG‐3′.

*Apoeb* forward 5′‐GGCAGTTTTAACTGGCTGCCAG‐3′.

*Apoeb* reverse 5′‐CCAGCCAGGAGCTGAAGATCTTTAC‐3′.

*Hexb* forward 5′‐CTTTGGGGAGAGTATGTGGACGC‐3′.

*Hexb* reverse 5′‐CAGGTATGCCTCTCCTGACCAT‐3′.

*P2ry12* forward 5′‐CTTCAGGTCGTCGCTGTTTA‐3′.

*P2ry12* reverse 5′‐AGTGCGTTTCCCTGTTGAT‐3′.

*Csf1ra* forward 5′‐CCTGATCCGCAACGTTCATCCT‐3′.

*Csf1ra* reverse 5′‐GCTTTGGGCAGCATTCTTGAGG‐3′.

*Ipcat2* forward 5′‐CTGGAGAAGGTTTCTGCACAGAAGAG‐3′.

*Ipcat2* reverse 5′‐CCTGCCAAAGATTGGTGCTTCTAG‐3′.

*Parvg* forward 5′‐GGAGCGTTAAACTCATTCACAGCAG‐3′.

*Parvg* reverse 5′‐GGGACATTCCTTGTTAGACTCTCTGTC‐3′.

*Plnxb2a* forward 5′‐GCACCCTGAGAGTGGTTCTCTAC‐3′.

*Plnxb2a* reverse 5′‐GTGACTGAGATGCGTCCGTTCATT‐3′.

*Irf8* forward 5′‐GACCTCTCAATGCTGCTGTTGTTC‐3′.

*Irf8* reverse 5′‐CGCTCATTCTTAATGCCGTCAATGG‐3′.

*Mpeg1.1* forward 5′‐GGGTTCAAGTCCGTAACCATCTGTAC‐3′.

*Mpeg1.1* reverse 5′‐CTTCTTGCACCAATGTGGCTCC‐3′.

*Spi1b* forward 5′‐CATCATCCCACCCAAAGAAGAGGG‐3′.

*Spi1b* reverse 5′‐CATGTAGTGACTGCACGCTTTGTAG‐3′.

Melting curve analysis was used to ensure primer specificity. For qPCR analysis, the threshold cycle (Ct) values for each gene were normalized to expression levels of ß‐actin and relative quantification of gene expression determined with the comparative Ct (ΔΔCt) method using the LightCycler® 96 Software (Roche). qPCR analysis was performed in triplicate for each gene.

### 2.9 Statistical analysis

Statistical analysis for qPCR and measurements of median fluorescence intensity of microglia were performed as followed. All experiments were performed in three replicates. All measured data were analyzed (StatPlus, AnalystSoft Inc.). One‐way ANOVA with Bonferroni's post hoc test was performed for comparisons between multiple experimental groups. Statistical values of *p* < .05 were considered to be significant. All graphs were plotted in Prism 6.1 (GraphPad Software) and values presented as population means ± *SD*.

## 3 RESULTS

### 3.1 Larval zebrafish microglia show a rapid differentiation

**Figure 1:**
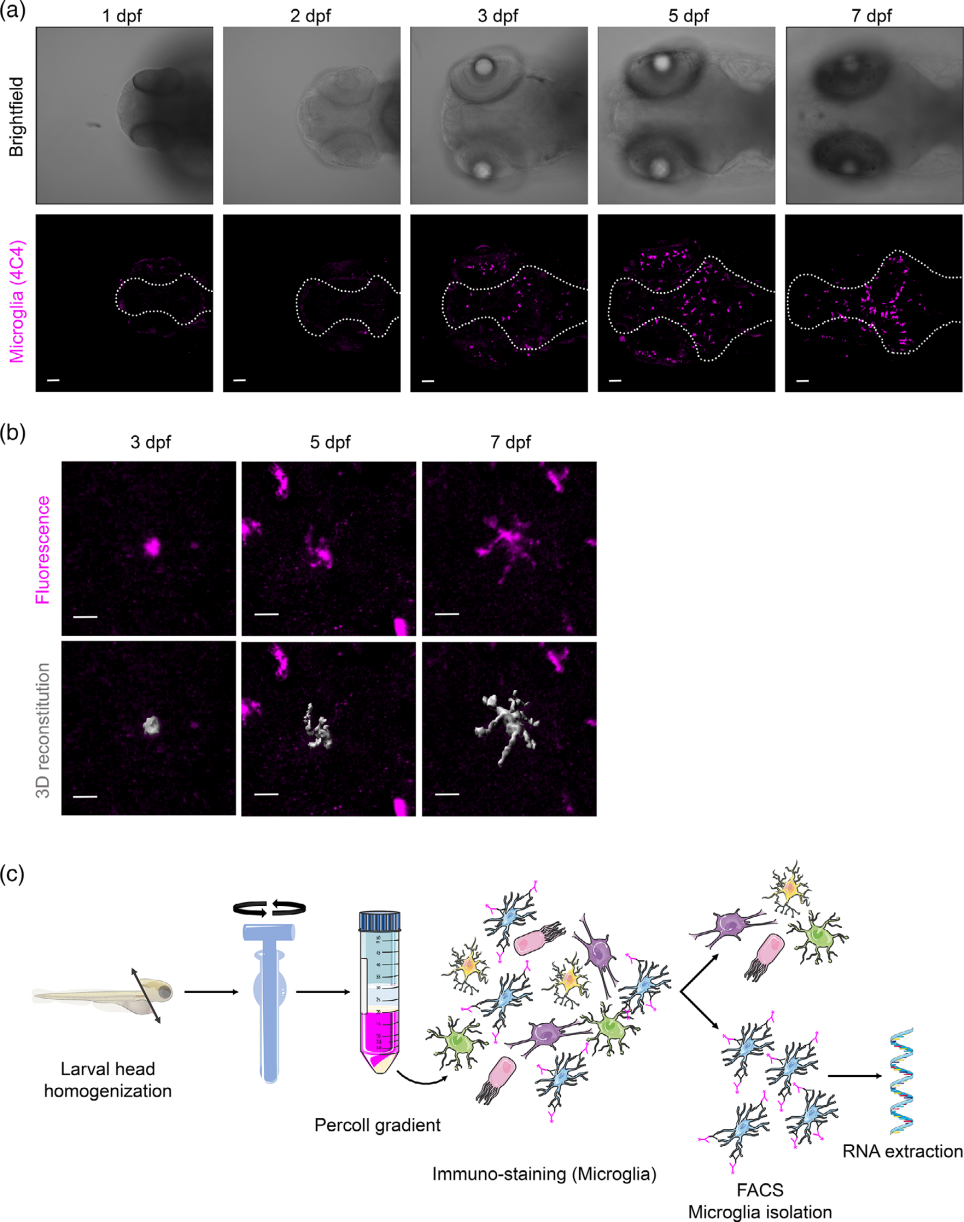
Development of the microglia population in larval zebrafish. (a) Representative confocal images are shown to illustrate zebrafish larval head development from 1 to 7 days postfertilization (dpf) and microglial cell distribution throughout the developing brain. Upper panels correspond to a brightfield transmission image and lower panels represent the maximum intensity projection of 4C4^+^ microglia (magenta) at each developmental stage. Microglia start colonizing the brain (dotted line) at 3 dpf whereas signal can be detected in the retina from 1 dpf onwards. Scale bar represents 50 μm. (b) Upper panels show 4C4 antibody immunohistochemistry, lower panels show segmented images of microglia morphology at 3, 5, and 7 dpf using the Imaris surface tool. Microglia morphology changes from amoeboid (1 dpf) to ramified (7 dpf) with an intermediate feature at 5 dpf. Scale bar represents 10 μm. (c) Schematic representation of the protocol used to isolate 4C4^+^ microglia from larval zebrafish brains at 3, 5, and 7 dpf. All images represent maximum intensity projections of confocal stacks. Images were captured using a Zeiss LSM710 confocal microscope with a 20×/NA 0.8 objective [Color figure can be viewed at http://wileyonlinelibrary.com]

**Figure 2:**
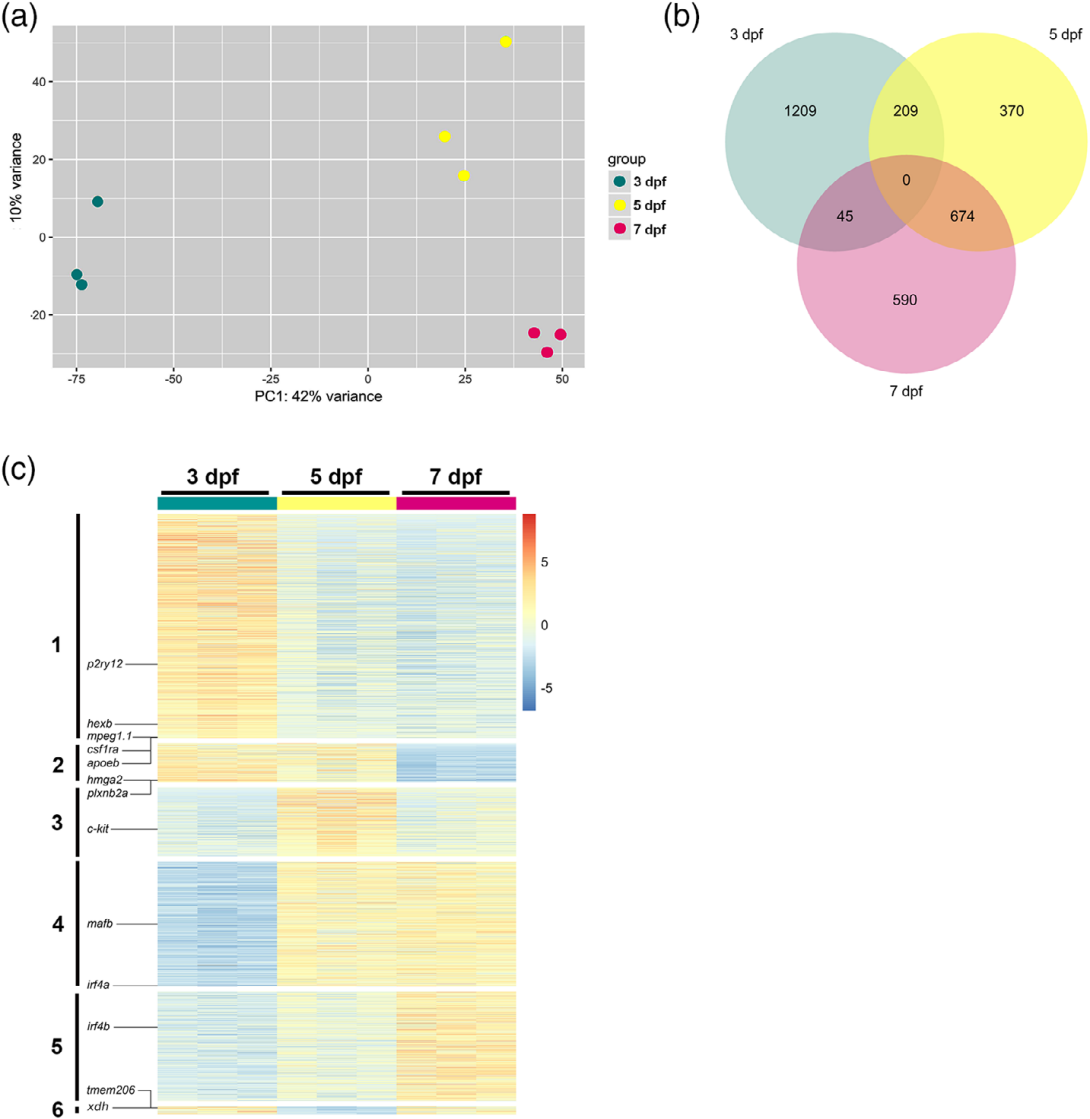
Zebrafish microglia transcriptome at 3, 5, and 7 dpf. (a) Principal component analysis (PCA) score plot obtained from normalized transformed read counts of isolated microglia RNA from 600 zebrafish embryos at 3 (green), 5 (yellow), and 7 (magenta) dpf, *n* = 3. The PCA score plot shows that replicates from 3, 5, and 7 dpf are clustered and separated according to their developmental stages. (b) Venn diagram shows unique and intersecting genes (3,097) differentially expressed (DE) from microglia transcriptome at 3, 5, and 7 dpf (FDR < 0.05, Fold Change > |2|). (c) Heatmap of DE genes from microglia transcriptome comparisons between 3, 5, and 7 dpf. This heatmap reveals six groups of different expression profiles. These groups correspond to the groups shown in the Venn diagram in (b), which provides the number of genes within these groups. See also Table S1. FDR, false discovery rate [Color figure can be viewed at http://wileyonlinelibrary.com]

**Figure 3:**
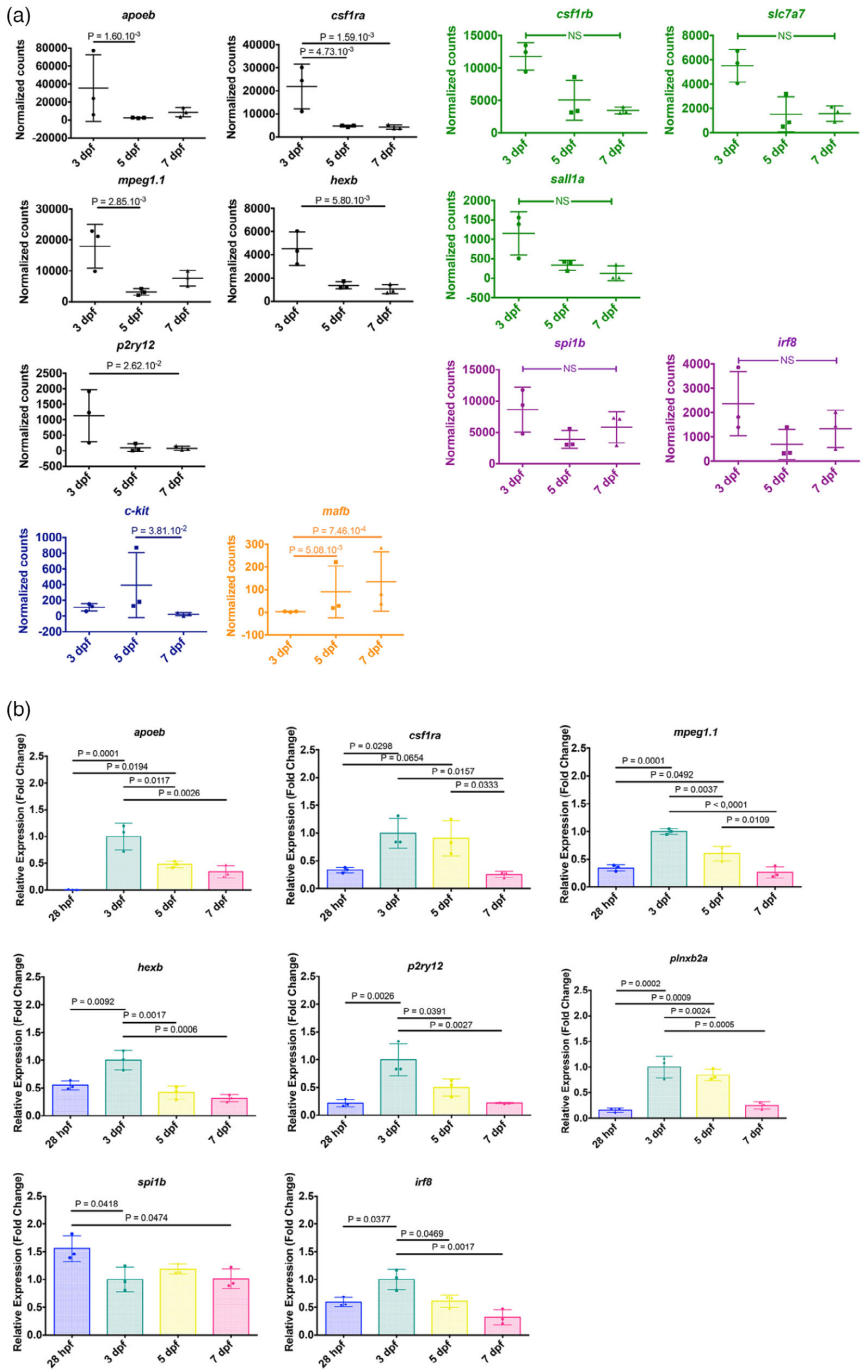
Larval zebrafish microglia show expression of microglia core signature genes. (a) Dot plots of normalized transformed read counts of a representative set of microglia genes (black; orange) and erythro‐myeloid progenitor (EMP) genes (blue) that show significant differences during development and dot plots of normalized transformed read counts of a representative set of microglia (green) and macrophage (purple) genes that show no significant differences between 3, 5, and 7 dpf. The means ± SD of three independent experiments are plotted. (b) mRNA expression levels for *apoeb*, *csf1ra*, *mpeg1.1*, *hexb*, *p2ry12*, *plnxb2a*, *spi1b*, and *irf8* from isolated macrophages at 28 hpf and microglia at 3, 5, and 7 dpf determined by qPCR (*n* = 3 for each gene). Fold change was measured in relation to 3 dpf microglia using the comparative (ΔΔCT) method. The means ± SD of three independent experiments are plotted [Color figure can be viewed at http://wileyonlinelibrary.com]

To understand the changes in gene expression during development in larval zebrafish microglia, we isolated microglia at three different time points. We chose 3 dpf, when macrophages start colonizing the brain and differentiate into early microglia (Figure 1a,b), 5 dpf when differentiation based on morphological criteria (ramification) is apparent (Figure 1a, b) and 7 dpf when microglia differentiation has further proceeded (Figure 1a, b). Microglia were isolated from dissociated brains using the microglia‐specific 4C4 antibody to perform immunohistochemistry followed by fluorescence‐activated cell sorting (FACS) (Figure 1c, Figure S1; Mazzolini et al., 2018). As shown in Figure 1 and in recent publications, the 4C4 antibody is highly specific for zebrafish microglia and does not detect other cell types in the brain (Chia, Mazzolini, Mione, & Sieger, 2018; Ohnmacht et al., 2016; Tsarouchas et al., 2018). To confirm the purity of the sorted 4C4+ cell population, we isolated cells from two different transgenic backgrounds with either labeled neurons (NBT:dsRED) or labeled radial glial cell progenitors (zic4:mCherry; Distel et al., 2009; Peri & Nüsslein‐Volhard, 2008). Analysis of the 4C4+ cells from these backgrounds revealed that 11.3% of the 4C4+ cells were positive for the neuronal marker and 12.6% were positive for the radial glia cell marker (Figure S2). Closer inspection of these cells via confocal microscopy revealed that the neuronal and radial glial cell signals were exclusively detected within the 4C4+ cells (Figure S2). We conclude that these cells represent microglia that had phagocytosed neurons and radial glial cell progenitors. This is in line with the high phagocytic activity of microglia during larval zebrafish development (Casano et al., 2016; Mazaheri et al., 2014; Peri & Nüsslein‐Volhard, 2008). Thus, we were confident to specifically isolate microglia via 4C4 immunohistochemistry followed by FACS. For each time point, microglia were pooled from 600 larval zebrafish brains and three biological replicates (600 brains per replicate) were performed per time point for RNA sequencing. Scatter plots of the read counts showed that despite the fact that each sample consisted of microglia from 600 brains, biological replicates were highly correlated (*r* > .8, Figure S3). Principal component analysis (PCA) confirmed this correlation by showing clusters corresponding to the three time points with 7 and 5 dpf samples appearing closer together compared to the 3 dpf samples (Figure 2a). We detected a large number of differentially expressed (DE) genes between the three developmental stages and focused on 3,097 of the most significant genes throughout development (False discovery rate [FDR] < 0.05, Fold Change > |2|, Figure 2b,c, Table S1). These genes were divided into six groups that showed differential expression through the three developmental stages (Figure 2c). The first group of genes showed higher expression at 3 dpf compared to 5 and/or 7 dpf (Figure 2c, 1,209 genes in total (Figure 2b, Table S1). This group contained typical microglia genes including *apoeb*, *p2ry12*, *hexb*, *csf1ra*, and *mpeg1.1* (Figures 2c and 3a, black). This is in line with previous descriptions of a rapid differentiation into early microglia in zebrafish at 3 dpf (Herbomel et al., 2001). The second group of genes were higher expressed at 3 and 5 dpf compared to 7 dpf (Figure 2c, 209 genes in total; Figure 2b, Table S1). Within this group, we did not detect any of the typical microglial genes. In this group, we detected for example, *plxnb2a*, which is one of the zebrafish orthologs for Plexin‐B2. Interestingly, Plexin‐B2 has been shown to negatively regulate motility in macrophages (Roney et al., 2011). As zebrafish microglia show a reduced motility at later developmental timepoints, the observed lower expression levels might suggest a conserved role for *plxnb2a*. Another gene detected in group 2 was *hmga2*, which has recently been shown to be a driver of inflammation in murine macrophages (Huang et al., 2017). Interestingly, Hmga2 shows high expression levels in murine microglia compared to other brain cells as well (Zhang et al., 2014). The third group showed higher expression levels on 5 dpf in comparison with 3 and/or 7 dpf (Figure 2c, 370 genes in total (Figure 2b, Table S1). Interestingly, the myeloid progenitor marker *c‐kit* appeared in this group (Figures 2c and 3a, blue). The fourth group showed lower expression levels on 3 dpf compared to 5 and 7 dpf (Figure 2c), 674 genes in total (Figure 2b, Table S1). Importantly, the gene *mafb*, recently identified as a marker for adult mouse microglia (Matcovitch‐Natan et al., 2016), followed the expression pattern of this group (Figure 2c and 3a, orange). This group also included one of the zebrafish orthologs of *interferon regulatory factor 4* (*irf4a*), which has been shown to be involved in microglia differentiation and polarization (Nam & Lim, 2016). The fifth group contained the genes with higher expression at 7 dpf in comparison to 3 and/or 5 dpf and included the other zebrafish *irf4* ortholog (*irf4b*; Figure 2c, 590 genes in total (Figure 2b, Table S1). Finally, the sixth group showed a small number of genes with higher expression levels at 3 and 7 dpf compared to 5 dpf (Figure 2c, 45 genes in total (Figure 2b, Table S1). Within this group, we detected genes that have been shown to be involved in inflammasome activation in macrophages. For example, xanthine dehydrogenase (XDH) has been shown to be converted to xanthine oxygenase (XH) upon oxidative stress and to regulate IL1b secretion upon inflammasome activation in macrophages (Ives et al., 2015). Interestingly, Xdh also shows higher expression levels in murine microglia compared to other brain cells (Zhang et al., 2014). Furthermore, we detected *tmem206* within this group. *tmem206* codes for a proton‐activated chloride channel, which has recently been shown to be involved in acid‐induced cell death (Yang et al., 2019). As for Xdh, the murine version of Tmem206 shows higher expression levels in microglia compared to other brain cells (Zhang et al., 2014).

In addition to the genes that showed significant differences in their expression levels at the three developmental stages (Figure 2c), we looked at the expression levels of other typical microglia genes including *slc7a7*, *sall1a*, and *csf1rb* (Figure 3a, green). Indeed, we detected these genes with slight differences in their expression levels at 3, 5, and 7 dpf (not significant FDR > 0.05; Figure 3a, green).

We also observed macrophage lineage genes at the three selected stages of zebrafish development such as *irf8* and *spi1b*, which have been shown to be crucial for microglia development (Kierdorf et al., 2013; Figure 3a, purple). Also, these genes showed slightly higher expression levels at 3 dpf (not significant FDR > 0.05) and appeared to be relatively stable at later time points (Figure 3a, purple).

Several of the typical macrophage and microglia marker genes showed higher expression levels at 3 dpf compared to 5 and/or 7 dpf, which implies an upregulation of these genes during early microglial differentiation. Thus, we decided to compare expression levels of these genes to primitive macrophages, the microglia progenitors in larval zebrafish. To do so, we isolated primitive macrophages at 28 hpf from transgenic mpeg1:EGFP larvae in which all macrophages are labeled with eGFP (Ellett et al., 2011). Macrophages were isolated from entire larvae and purified based on their eGFP expression via FACS (Figure S4). Additionally, we isolated microglia at 3, 5, and 7 dpf based on 4C4 expression as described before. qPCR was performed to compare expression levels of genes of interest. Importantly, we detected significantly higher expression levels for microglial genes such as *apoeb*, *p2ry12*, *hexb*, and *csf1ra* in microglia at 3 dpf compared to macrophages at 28 hpf (Figure 3b). *irf8*, *mpeg1.1*, and *plxnb2a* showed a similar trend while *spi1* showed higher expression levels in macrophages at 28 hpf compared to microglia at 3 dpf (Figure 3b). These results further support our conclusion that microglia specific genes are strongly upregulated during the initial steps of microglial differentiation at 3 dpf. Furthermore, the qPCR results validate the observed expression changes between 3, 5, and 7 dpf observed in the RNA sequencing analysis.

**Figure 4:**
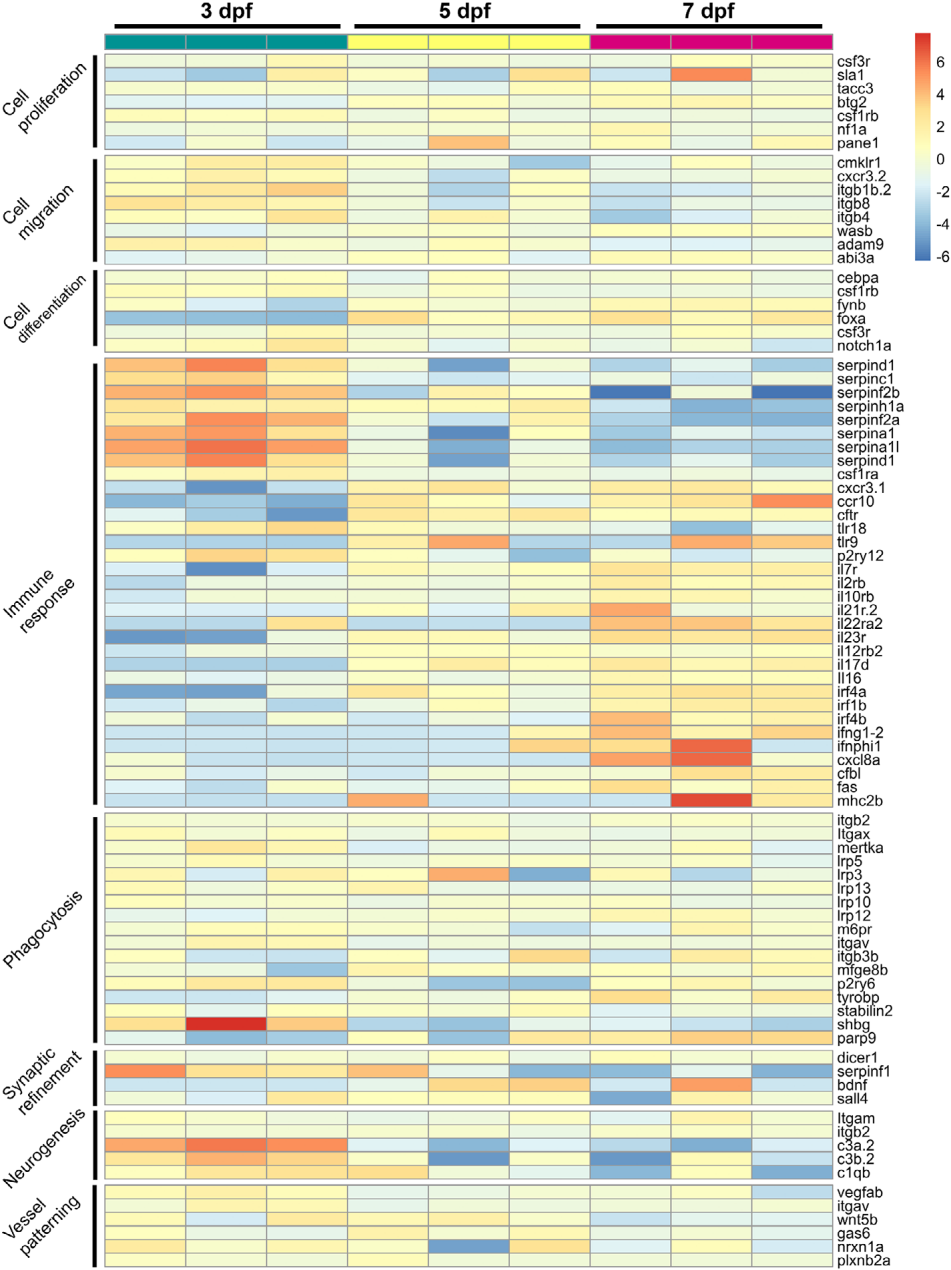
Expression profile of genes involved in microglial processes during development. Heatmap representing normalized transformed read counts of 87 genes involved in different microglia processes at 3, 5, and 7 dpf [Color figure can be viewed at http://wileyonlinelibrary.com]

After the global analysis of gene expression changes, we specifically focused on genes that are involved in processes required for invasion of macrophages into the brain, differentiation into microglia, and microglial functions (Table S2). These genes were selected according to their classification into the different GO categories (cell proliferation, cell migration, cell differentiation, immune response, neurogenesis, synaptic refinement, and vessel patterning) or due to specific roles described in macrophages/microglia (phagocytosis; Brown & Neher, 2012; Lemke, 2019). Interestingly, while genes involved in proliferation showed a slight trend toward increased expression from Day 5, we detected the contrary for genes involved in migration, which showed higher expression levels at 3 dpf and lower expression levels from 5 dpf (Figure 4). Genes involved in differentiation showed opposite trends, with *csf1rb*, for example, showing highest expression levels at 3 dpf and *foxa* showing higher expression levels from 5 dpf (Figure 4). Interestingly, genes involved in immune responses showed different profiles as well. The family of serpin genes, for example, showed higher expression levels at 3 dpf compared to 5 and 7 dpf, while most other immune genes showed higher expression levels toward the later developmental stages (Figure 4). Genes involved in phagocytosis showed relative constant expression levels throughout development (Figure 4). Furthermore, microglial genes that have been shown to be involved in synaptic refinement and vessel patterning showed constant expression levels, while genes involved in neurogenesis showed higher expression at 3 dpf (Figure 4).

**Figure 5:**
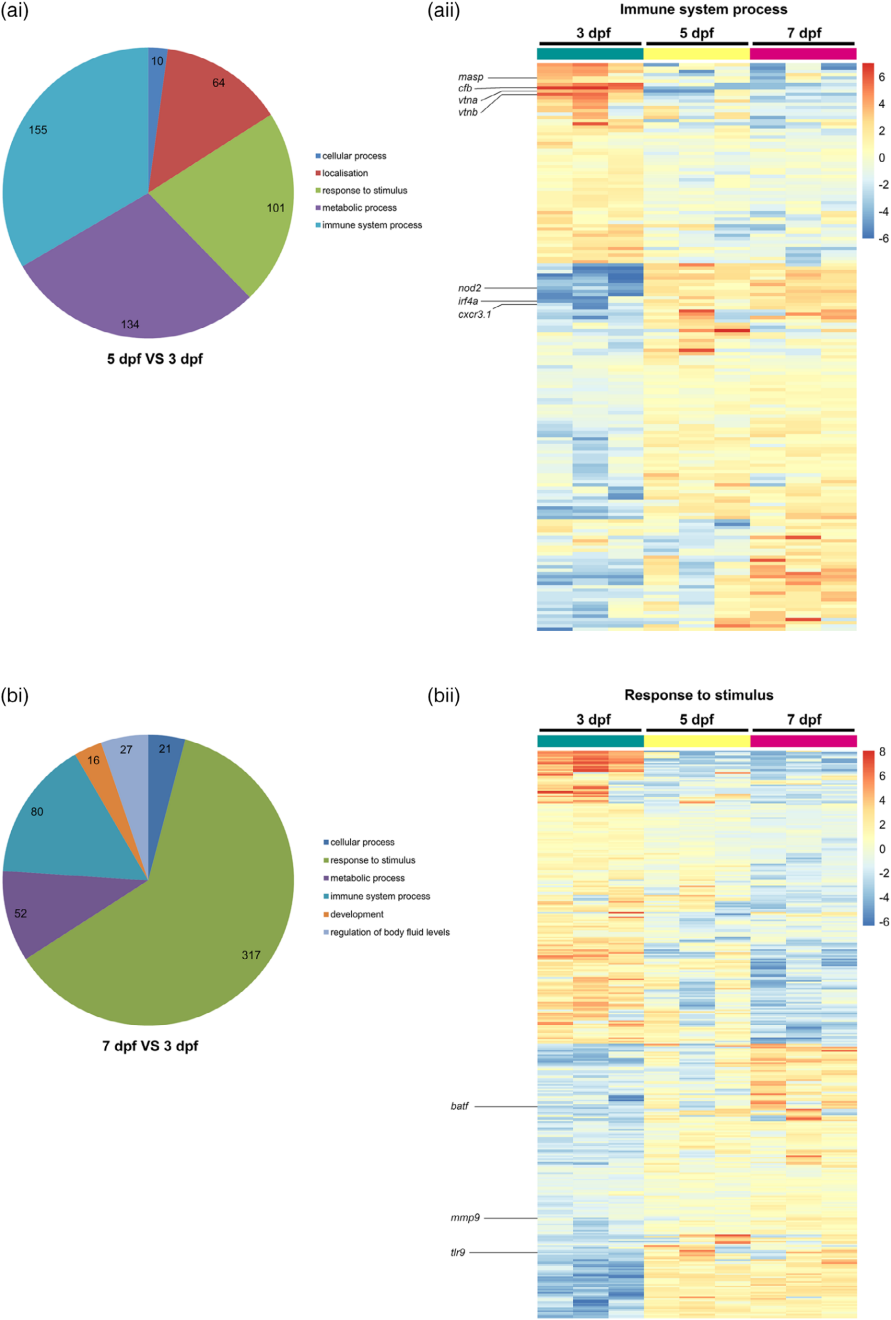
Gene Ontology (GO) categories analysis of DE larval zebrafish microglia genes. (a‐i) Pie chart representation for significant enrichment of GO for DE genes from microglia transcriptome comparison between 5 and 3 dpf (FDR < 0.05, fold change > |2|). Only categories containing at least 10 genes are represented. (a‐ii) Heatmap of DE genes belonging to the GO category “immune system process” containing the highest number of genes (155) from microglia transcriptome comparison between 5 and 3 dpf. (b‐i) Pie chart representation for significant enrichments GO for DE genes from microglia transcriptome comparison between 7 and 3 dpf (FDR < 0.05, fold change > |2|). Only categories containing at least 10 genes are represented. (b‐ii) Heatmap of genes belonging to the GO category “Response to stimulus” containing the highest number of genes (317) from microglia transcriptome comparisons between 7 and 3 dpf [Color figure can be viewed at http://wileyonlinelibrary.com]

In order to get a full overview on the biological processes that showed significant changes in different developmental stages, we performed an enrichment analysis with the GO database using Gorilla (for entire analysis see Tables S3 and S4). Thus, we analyzed the distribution of the 3,097 identified differentially expressed genes between the three developmental stages (Figure 2, Table S1). Among the significant categories, “immune system process” was the most represented between 3 and 5 dpf followed by “metabolic process” and “response to stimulus” (Figure 5a‐i). In contrast, the trend was inverse for 7 dpf versus 3 dpf and the differentially expressed genes belonging to “response to stimulus” were largely represented followed by “immune system process” and “metabolic process” (Figure 5b‐i). Furthermore, new categories appeared at the 7 dpf stage such as “cellular process”, “regulation of body fluid levels” and “development” (Figure 5b‐i). No category was significantly overrepresented in the comparison of 7 dpf versus 5 dpf (data not shown).

As the categories, “immune system process” and “response to stimulus” were the most strongly represented, we analyzed these categories in more detail. We obtained 173 genes for the “immune system process” that were differentially expressed between the three stages of development and we identified 366 genes for the response to stimulus category (Figure 5a‐ii,b‐ii). Interestingly, as described before, the strongest changes in gene expression were observed between 3 and 5 dpf. Among the genes in the category “immune system process,” the majority of genes were significantly upregulated between 3 and 5 dpf (Figure 5a‐ii). Among these genes, we detected intracellular pathogen receptors such as *nod2*, chemokine receptors such as *cxcr3.1* but also *irf4a*, which is not only involved in differentiation but also regulates anti‐inflammatory genes in macrophages/microglia (Nam & Lim, 2016). In contrast, we detected another set of genes that was strongly expressed at 3 dpf and significantly downregulated from 5 dpf (Figure 5a‐ii). These genes include for example *vitronectin* (*vtna*, *vtnb*), important for microglia activation and phagocytosis of dead cells (Welser‐Alves & Milner, 2013) as well as genes involved in the complement cascade such as *complement factor B* (*cfb*) and *mannan‐binding lectin serine peptidase 2* (*masp2*; Figure 5a‐ii).

Within the “response to stimulus” category, approximately half of the genes showed higher expression levels at 3 dpf and significant downregulation from 5 dpf, while the other half of genes were lower expressed at 3 dpf and increased expression from 5 dpf (Figure 5b‐ii). Interestingly, among the genes with increased expression from 5 dpf, we detected *toll‐like‐receptor 9* (*tlr9*), which has been recently implicated in sensing self‐DNA from degenerating neurons and the microglia‐mediated attenuation of aberrant neurogenesis (Matsuda et al., 2015). Levels of *matrix metallopeptidase 9* (*mmp9*) were also elevated from 5 dpf compared to 3 dpf, which might be in line with the recently described role for *mmp9* in the development of sensory circuits (Reinhard, Razak, & Ethell, 2015; Figure 5b‐ii). Furthermore, we detected increased levels of *basic leucine zipper ATF‐like transcription factor* (*batf*), which appears to be a microglia‐specific transcription factor that is not expressed in other brain cells (Zhang et al., 2014; Figure 5b‐ii).

In summary, this analysis shows that although several microglia‐specific genes are already strongly expressed at 3 dpf, there is a large number of gene expression changes between 3 and 5 dpf. This implies that the differentiation of microglia during zebrafish development takes place mainly between 3 and 5 dpf, which is in line with morphological observations showing that from 5 dpf zebrafish microglia start exhibiting a reduced motility and an increase in ramification (Svahn et al., 2013). The changes between 5 and 7 dpf seem to be minor, which is also supported by the close clustering of the 5 and 7 dpf samples in the PCA (Figure 2a). These changes may represent the final adaptations to the neural environment.

### 3.2 Larval zebrafish microglia show differences in gene regulation compared to developing mouse microglia

**Figure 6:**
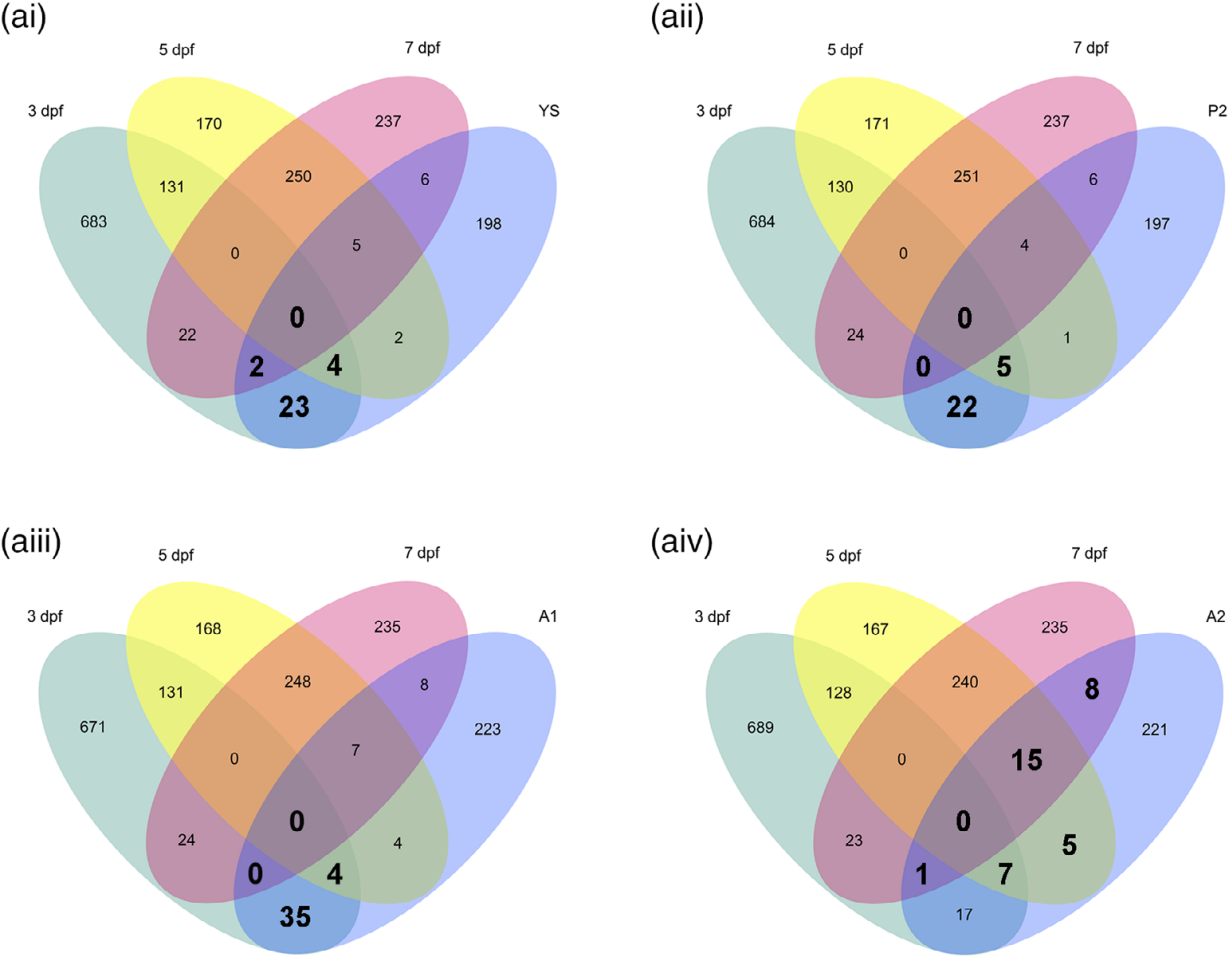
Differential gene expression during microglia development in the larval zebrafish shows differences to microglia development in the mouse. Venn diagrams showing unique and intersecting DE genes from zebrafish microglia transcriptome at 3 (green), 5 (yellow), and 7 (magenta) dpf and DE genes from mouse microglia (blue) in the (Y) yolk sac (a‐i), in the (P2) postnatal stage 2 (a‐ii), in the (A1) adult stage 1 (a‐iii) and in the (A2) adult stage 2 (a‐iv, FDR < 0.05, Fold Change > |2|). Mouse data obtained from Matcovitch‐Natan et al. (2016). Significant gene enrichments are shown in bold (FDR < 0.05) [Color figure can be viewed at http://wileyonlinelibrary.com]

As we detected groups of differentially regulated genes during development of zebrafish microglia, we decided to test if the regulation of these genes is evolutionarily conserved. Thus, we compared the identified DE genes to differentially regulated genes during mouse microglia development. To this aim, we used the expression data for mouse microglia from Matcovitch‐Natan et al. (2016). The authors identified 3,059 differently expressed genes that showed dynamic changes in microglia throughout development by comparing four stages: (a) Embryonic microglia in the yolk sac and (b) in the brain, (c) the microglia post birth, and (d) finally the adult microglia. We accessed the data from Matcovitch‐Natan et al. and processed and normalized their data together with our samples. Then we compared the expression of larval zebrafish microglia at 3, 5, and 7 dpf to the different stages of the Matcovitch‐Natan et al. study. This comparison revealed a strong correlation of the larval zebrafish microglia gene expression and the microglial gene expression during mouse development (*r* ~ .6, Table S5). Additionally, Matcovitch et al performed a clustering analysis and identified seven gene expression clusters corresponding to the substages of development. Matcovitch‐Natan et al identified seven gene expression clusters corresponding to the stages of development. These clusters are YS for yolk sac (373 genes), E for early microglia (including E1 and E2 corresponding to day E10.5 and E14 (1,289 genes), P for premicroglia (including P1 and P2 corresponding day E14 and postnatal Day 9 [P9], 589 genes) and A for adult microglia (clusters A1 and A2, 4 weeks and onward; 808 genes). Out of the 3,059 mouse genes in total, we identified 2,086 annotated zebrafish orthologs. To test if the same genes show dynamic regulation in zebrafish and mice during microglial development, we compared these genes with the differentially expressed genes in larval zebrafish microglia at 3, 5, and 7 dpf (Table S6). To do this, we performed an enrichment analysis between the significant genes identified in mouse and the genes differentially expressed in developmental zebrafish microglia (3, 5, and 7 dpf; FDR < 0.05, Fold Change > |2|). The significance of enrichment was calculated using a hypergeometric distribution. First, we looked at genes differentially expressed in zebrafish microglia at 3 dpf. We observed significant enrichment between the genes differentially expressed in zebrafish microglia at 3 dpf and those expressed in mouse microglia in the yolk sac (29 genes in common, fold enrichment = 1.81, *p*‐value = 1.35 × 10^−3^, Table S6) as well as in the postnatal stage P2 and the adult stage A1 (27 and 39 genes in common, fold enrichment = 1.72 and 2.08, *p* values = 9.71 × 10^−4^ and 1 × 10^−5^, respectively, Table S6; Figure 6). The comparison to the adult stage A2 was not significant and revealed a slightly lower number of shared genes (25 genes, *p*‐value = 6.85 × 10^−2^, Table S6; Figure 6). Next, we looked at genes differentially expressed in zebrafish microglia at 5 dpf. The comparison of differentially expressed genes in zebrafish microglia at 5 dpf and the dynamically regulated mouse microglia genes revealed a different pattern. Here we observed only a low number of shared genes with the yolk sac, the postnatal stage P2 and the adult stage A1 microglia (11, 10, and 15 genes in common, *p* values = .47, .57, and .24, respectively; Figure 6). However, we observed a significant enrichment with 27 shared genes between the genes significantly higher expressed in zebrafish microglia at 5 dpf and those expressed in the adult A2 microglia in the mouse (Figure 6, fold enrichment = 2.27, *p*‐value = 5.91 × 10^−5^, Table S6). Finally, we looked at genes differentially expressed in zebrafish microglia at 7 dpf. The comparison of zebrafish microglia genes significantly higher expressed at 7 dpf showed only 13 shared with the yolk sac stage, 10 shared genes with the postnatal stage P2, but 15 shared genes with the in adult stage A1 and 24 shared genes with the adult A2 stage in the mouse (Figure 6, fold enrichment = 2.18, *p*‐value = 2.96 × 10^−4^, Table S6). GO term analysis of the shared and different genes between the 3, 5, and 7dpf zebrafish microglia and the mouse microglia in the different developmental stages revealed that most GO terms showed an equal frequency for shared as well as different genes (Table S7). However, we observed differences for some categories which were only represented by either the zebrafish specific genes, the mouse‐specific genes or the shared genes (Table S7). For example, the category response to stress was only represented by zebrafish specific genes in the comparison 3 dpf to mouse P2 and A2 (Table S7). On the contrary, the categories cellular component assembly and transmembrane transport were only represented by mouse‐specific genes in the comparison 3 dpf to mouse P2 and A2 (Table S7). Interestingly, the category cell proliferation was only represented by shared genes in the comparison 5 and 7 dpf to mouse A2 microglia (Table S7). Details on the distribution across the different categories can be found in Table S7 and the list of all shared genes is presented in Table S6.

In summary, this analysis shows that 3 dpf larval zebrafish microglia show an enrichment of genes that are also dynamically regulated throughout microglia development in the mouse. In contrast, 5 and 7 dpf larval zebrafish microglia mainly exhibit more similarities in regulation of gene expression with adult (A2) mouse microglia.

### 3.3 Larval zebrafish microglia share similarities with adult zebrafish microglia

In the zebrafish, larval microglia are replaced by a second wave of hematopoietic stem cell‐derived cells, which give rise to the adult microglia population. To test if the gene expression in larval zebrafish microglia is comparable to the gene expression in adult zebrafish microglia, we compared our data set to the previously published adult zebrafish microglia transcriptome (Oosterhof et al., 2016). We accessed the original data from Oosterhof et al. and processed their data together with our 3, 5, and 7 dpf microglia samples. To compare the gene expression globally, we estimated the correlation between adult zebrafish microglia and larval 3, 5, and 7 dpf microglia. This analysis revealed a high correlation for all time points (*r* = .79 for 3 and 5 dpf, *r* = .82 for 7 dpf, Table S5). Thus, we decided to directly compare the gene expression between adult microglia and the different developmental time points. To do this, we first compared the gene expression of microglia at 3, 5, and 7 dpf with the gene expression of other brain cells (from Oosterhof et al., 2016) and identified genes that are differentially expressed in larval microglia (FDR < 0.05, fold change >2). This analysis revealed differential expression for 2,606 genes at 3 dpf, 2,645 genes at 5 dpf, and 2,778 genes at 7 dpf in larval microglia and a clear separation of microglia‐specific genes from other brain cells as confirmed by PCA plot (Figure S5, Table S8). We then compared these genes to the differentially expressed genes in adult zebrafish microglia (2,589 genes) and a hypergeometric distribution was used to compute the significance of enrichment.

**Figure 7:**
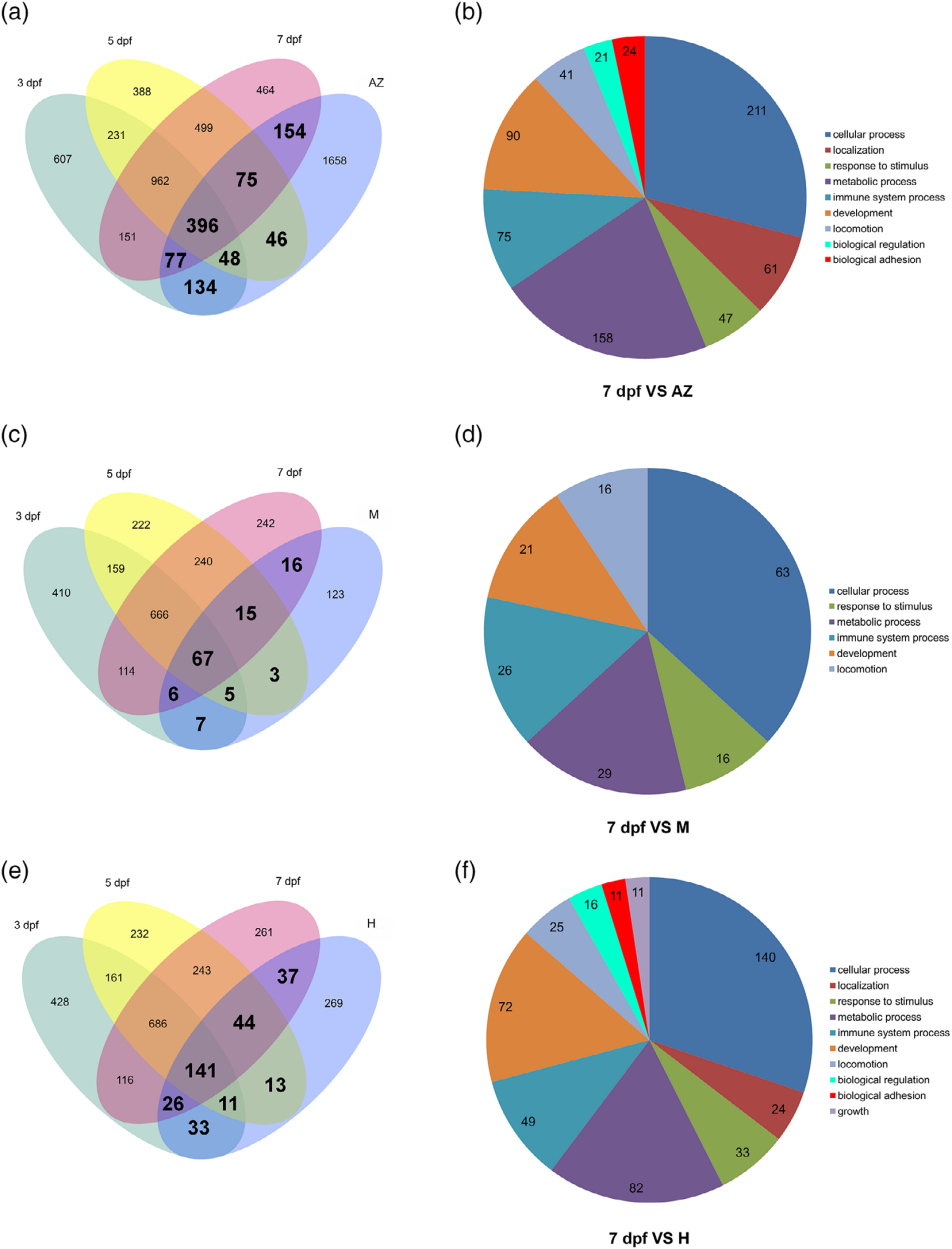
Larval zebrafish microglia share a significant number of DE genes with adult zebrafish microglia, adult mouse microglia, and human microglia. (a) Venn diagram showing unique and intersecting DE genes from zebrafish microglia transcriptome at 3 (green), 5 (yellow), and 7 (magenta) dpf versus other brain cells in comparison to DE genes from adult zebrafish (AZ) microglia transcriptome versus other brain cells (Oosterhof et al., 2016; FDR < 0.05, Fold change >2). Significant gene enrichments are shown in bold (FDR < 0.05). (b) Pie chart representation of GO categories for higher expressed genes in 7 dpf versus adult zebrafish microglia. (c) Venn diagram showing unique and intersecting DE genes from zebrafish microglia transcriptome at 3 (green), 5 (yellow), and 7 (magenta) dpf versus other brain cells in comparison to DE genes from adult mouse (M) microglia transcriptome versus other brain cells (Zhang et al., 2014). Significant gene enrichments are shown in bold (FDR < 0.05). (d) Pie chart representation of GO categories in 7 dpf zebrafish versus adult mouse microglia. (e) Venn diagram showing unique and intersecting DE genes from zebrafish microglia transcriptome at 3 (green), 5 (yellow), and 7 (magenta) dpf versus other adult brain cells in comparison to DE genes from human (H) microglia transcriptome versus other adult brain cells (Galatro et al., 2017). Significant gene enrichments are shown in bold (FDR < 0.05). (f) Pie chart representation of GO categories for higher expressed genes in 7 dpf zebrafish versus adult human microglia [Color figure can be viewed at http://wileyonlinelibrary.com]

Indeed, we detected significant enrichments for the three stages of development and the adult zebrafish microglia. The strongest enrichment was detected for the 7 dpf stage with 702 shared genes with adult microglia, reflecting an enrichment of 2.12‐fold (*p*‐value = 2.71 × 10^−99^; Figure 7a, Table S9). This was followed by the 3 dpf stage with 655 shared genes with adult microglia, representing a 2.11‐fold enrichment (*p*‐value = 1.74 × 10^−90^) and finally 5 dpf with 565 shared genes with adult microglia, showing a 1.8‐fold enrichment (*p*‐value = 4.2 × 10^−50^; Figure 7a, Table S9).

In summary, these results show that larval microglia share a significant number of expressed genes with adult microglia and the 7 dpf microglia are closest to their adult counterparts.

GO category analysis of the genes shared between 7 dpf microglia and adult microglia revealed that the most represented categories were “cellular process,” “metabolic process,” “development,” and “immune system process” (Figure 7b), highlighting the importance of these processes in the acquisition of microglia identity. However, this analysis also highlights the differences between larval microglia and adult microglia. Based on the comparison of 7 dpf microglia and adult microglia, we identified 1,886 DE genes that are specific for adult zebrafish microglia and 2,076 DE genes that are only expressed in larval 7 dpf microglia. Interestingly, GO category analysis of the genes specific for adult and larval microglia revealed similar GO terms in the top 10 of the most represented categories. These categories were “cellular nitrogen compound metabolic process,” “anatomical structure development,” “biosynthetic process,” “signal transduction,” “cellular protein modification process,” “cell differentiation,” “transport,” and “response to stress” (Table S7). This suggests that these processes are in part fulfilled by genes that are specific for either larval or adult microglia. Thus, these cellular processes might be different in larval and adult zebrafish microglia. These putative disparities might be due to the differences in ontogeny of larval and adult zebrafish microglia or reflect adaptations to larval and adult brains.

### 3.4 Gene expression in larval zebrafish microglia shows similarities to adult mouse microglia

As we detected similarities between larval zebrafish microglia and adult zebrafish microglia, we decided to test to what extent the gene expression profile of larval zebrafish microglia was comparable to adult mouse microglia. To address this question, we first performed a gene expression correlation analysis between larval zebrafish 3, 5, and 7 dpf microglia and the microglia‐specific genes from the mouse identified by Zhang et al. (2014). Then, we compared the genes that we identified to be differentially expressed in zebrafish microglia at 3, 5, and 7 dpf (compared to other brain cells; Table S8) with the genes differentially expressed in adult mouse microglia (Zhang et al., 2014). The correlation analysis revealed a strong correlation between the larval zebrafish microglia gene expression and the adult mouse microglia gene expression with the 7 dpf zebrafish microglia showing the highest correlation (*r* = .57, Table S5). Out of 500 differentially expressed genes in mouse microglia, we found 242 annotated zebrafish orthologs. Interestingly, 85 of these orthologs were detected among the differentially expressed genes in 3 dpf zebrafish microglia (3.18‐fold enrichment, *p*‐value = 1.25 × 10^−23^), 90 were detected in the differentially expressed genes in 5 dpf zebrafish microglia (3.5‐fold enrichment, *p*‐value = 2.32 × 10^−28^) and 104 were found in the differentially expressed genes in 7 dpf microglia (4.08‐fold enrichment, *p*‐value = 1.38 × 10^−39^; Figure 7c, Table S10). We then analyzed GO categories for the 104 genes shared between 7 dpf zebrafish microglia and mouse microglia. Interestingly, the most represented categories were again “cellular process,” “metabolic process,” “immune system process,” and “development” (Figure 7d), which were previously detected when comparing 7 dpf zebrafish microglia to adult zebrafish microglia.

In conclusion, out of the 242 annotated zebrafish orthologs of adult mouse microglial genes we detect up to 43% in larval zebrafish microglia (7 dpf) and a strong overall correlation (*r* = .57). This is almost the same degree of conservation to mouse microglia as previously shown for adult zebrafish microglia (45% genes in common with mouse microglia; Oosterhof et al., 2016).

### 3.5 Larval zebrafish microglia share a significant number of genes with human microglia

In order to compare the gene expression profiles of zebrafish larval microglia and adult human microglia, we used the expression data of human microglia from Galatro et al. (2017). First, we performed a correlation analysis of the global gene expression between zebrafish 3, 5, and 7 dpf microglia and human microglia. This revealed a strong overall correlation with zebrafish 7 dpf microglia showing the highest correlation to the human microglia (*r* = .69, Table S5). Then, we compared the genes that we have identified to be differentially expressed in zebrafish microglia at 3, 5, and 7 dpf (compared to other brain cells, Table S7) to the 1,297 differentially expressed genes found in human microglia (Galatro et al., 2017). Out of the 1,297 DE genes in human microglia, we identified 574 annotated zebrafish orthologs. Interestingly, we observed enrichments between the genes expressed at 3, 5, and 7 dpf in zebrafish microglia and those expressed in human microglia (Figure 7e). We observed 211 shared genes between the genes expressed in zebrafish microglia at 3 dpf and those expressed in human microglia (2.67‐fold enrichment, *p‐*value = 3.81 × 10^−46^), 209 shared genes between the genes expressed in zebrafish microglia at 5 dpf and those expressed in human microglia (2.77‐fold enrichment, *p*‐value = 3.19 × 10^−48^) and finally 248 shared genes for zebrafish microglia at 7 dpf and human microglia (3.24‐fold enrichment, *p*‐value = 5.96 × 10^−74^) (Figure 7e, Table S11). Importantly, among shared genes between larval microglia and human microglia we detected several of the typical microglia genes including *irf8*, *spi1*, *csf1ra*, *csf1rb*, *mpeg1.1*, *slc7a7*, *p2ry12*, and *p2ry13*. Finally, to get a broader understanding of the biological processes that appear to be conserved between zebrafish larval 7 dpf microglia and human microglia, we performed an enrichment analysis with the GO database using Gorilla. This comparison revealed that among the significant categories the groups with the largest numbers of genes were again “cellular process,” “metabolic process,” “immune system process,” and “development” (Figure 7f).

**Figure 8:**
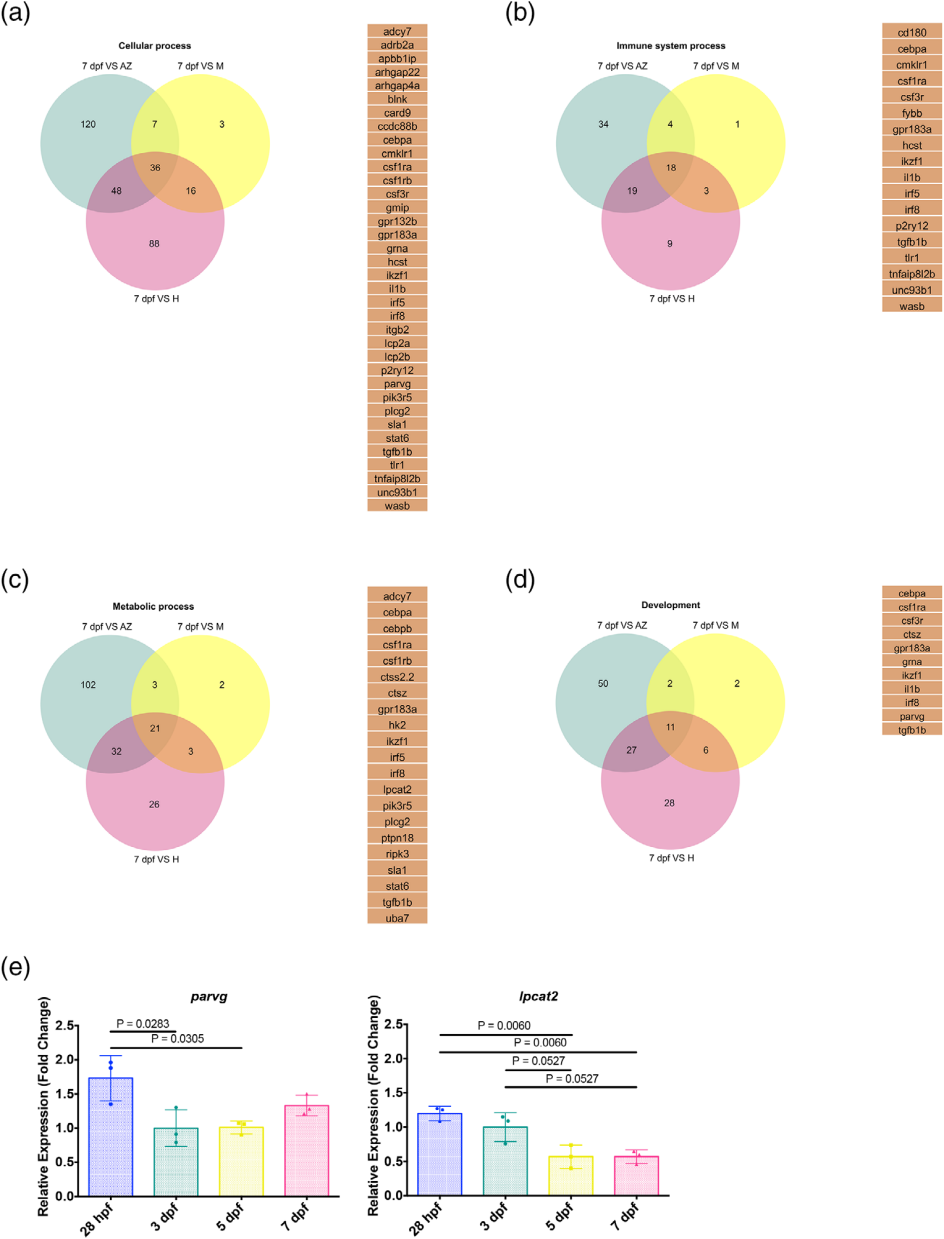
Conserved cellular, immune system, development, and metabolic process core genes across species. Venn diagrams showing unique and intersecting genes from 7 dpf zebrafish microglia transcriptome versus adult zebrafish microglia transcriptome (green), 7 dpf zebrafish microglia transcriptome versus mouse microglia transcriptome (yellow) and 7 dpf zebrafish microglia transcriptome versus human microglia transcriptome (magenta) belonging to the GO category “Cellular process” (a), “Immune system process” (b), “Metabolic process” (c), and “Development” (d). The core genes shared between zebrafish, mouse, and human for the different categories are listed. (e) mRNA expression levels for *parvg* and *lpcat2* from isolated macrophages at 28 hpf and microglia at 3, 5, and 7 dpf determined by qPCR (*n* = 3 for each gene). Fold change was measured in relation to 3 dpf microglia using the comparative (ΔΔCT) method. The means ± SD of three independent experiments are plotted [Color figure can be viewed at http://wileyonlinelibrary.com]

As these categories were also detected for the shared genes between larval 7 dpf microglia and adult zebrafish microglia and adult mouse microglia, respectively, we took a closer look at the genes within these categories. Thus, we compared the genes identified in the single comparisons and searched for genes that were shared among all. This comparison revealed a number of core genes for the different processes that are conserved across species. For the category “cellular process,” we identified 36 conserved genes, for “immune system” 18 genes, for the “metabolic process” 21 genes, and for development 11 genes (Figure 8). Among these genes, we detected some microglia specific genes such as *p2ry12* but also general macrophage genes such as *irf8* or *il1b*. As several of these genes have not been described in zebrafish microglia before, we decided to test if they were specific for microglia or expressed in macrophages as well. We selected *parvg* and *lpcat2* for qPCR comparison to 28 hpf primitive macrophages. Interestingly, *parvg* showed significantly higher expression levels in 28 hpf macrophages compared to microglia at 3 and 5 dpf, while *lpcat2* showed significantly higher expression levels in 28 hpf macrophages compared to microglia at 5 and 7 dpf (Figure 8e). Thus, these genes appear to be macrophage lineage genes rather than microglia specific in zebrafish.

In summary, the comparison to the human microglia transcriptome reveals a significant degree of conservation between larval zebrafish microglia and adult human microglia. Furthermore, a certain number of genes involved in a variety of processes appear to be evolutionarily conserved across species.

## 4 DISCUSSION

Larval zebrafish is a popular model to address the functions of innate immune cells in vivo. Over the past decade, a variety of transgenic lines has been established that allow the observation of neutrophils, macrophages, and microglia in the living larva. However, to gain an in‐depth understanding of their functions, there is a clear need to analyze their gene expression profiles. Here, we provide the first gene expression profiles of zebrafish microglia during larval development (3, 5, and 7 dpf). Importantly, our analysis showed that a large number of typical microglia genes is expressed in larval microglia. These genes include *apoeb*, *p2ry12*, *p2ry13*, *hexb*, *csf1ra*, *csf1rb*, *mpeg1.1*, *mafb*, *slc7a7*, and *sall1a*. Interestingly, while *csf1rb*, *slc7a7*, and *sall1a* showed relatively constant expression throughout development, other genes including *apoeb*, *p2ry12*, *hexb*, *csf1ra*, and *mpeg1.1* were more highly expressed at 3 dpf compared to 5 and 7 dpf. One explanation for this might be the fact that in zebrafish, primitive macrophages invade the larval CNS and undergo a rapid differentiation into microglia within 24 hr in zebrafish (Herbomel et al., 2001). This is supported by our qPCR analysis which shows rather low levels of expression for *apoeb*, *p2ry12*, *hexb*, and *csf1ra* in 28 hpf macrophages and a significant upregulation in 3 dpf microglia. Thus, expression of a subset of genes needed for the differentiation might be strongly upregulated during this period and normalized at later stages. The most significant changes in gene expression occurred between 3 and 5 dpf. GO analysis showed that significantly enriched terms were “immune system process,” “metabolic process,” “response to stimulus” but also “localization”. These changes probably reflect the full differentiation into microglia between 3 and 5 dpf. This is line with previous morphological observations describing the transition into the fully ramified microglial phenotype by 5 dpf in zebrafish larvae (Svahn et al., 2013). An interesting observation was the high expression of serpin family genes at 3 dpf of larval development. Serpin genes have been shown to be activated in microglia to counteract the toxic effects of thrombin upon breakdown of the blood‐brain barrier (Bedoui, Neal, & Gasque, 2018). We speculate that high levels of serpin genes might not be needed after 3 dpf, as the blood‐brain barrier is maturing at this developmental stage in zebrafish (Jeong et al., 2008).

Recent studies showed that larval microglia in zebrafish are replaced by a second wave of definitive microglia that persist throughout adulthood and are derived from cmyb‐dependent hematopoietic stem cells (Ferrero et al., 2018). Thus, to compare larval microglia and adult microglia, we compared our data set to the previously published adult zebrafish microglia transcriptome (Oosterhof et al., 2016). This comparison revealed a large number of shared genes expressed in larval and in adult zebrafish microglia (3 dpf: 655 genes, 5 dpf: 556 genes, 7 dpf: 702 genes). Importantly, among these genes, we detected many of the core microglia genes including *apoeb*, *p2ry12*, *hexb*, *csf1ra*, *csf1rb*, *mpeg1.1*, *slc7a7*, *irf8*, and *spi1*. GO analysis revealed that the common genes were mainly represented in the “cellular process,” “metabolic process,” “development,” and “immune system process” categories. The comparison of larval zebrafish microglia and adult zebrafish microglia also revealed a large number of genes that were specific for either larval or adult microglia. These differences may reflect adaptations to the larval and adult CNS or they might be inherited due to the different origins of larval and adult microglia. As Matcovitch‐Natan et al. (2016) detected significant differences in gene expression between embryonic and adult microglia in mice as well, it is tempting to speculate that the adaptation to the different brain environment might be the underlying cause. Future transplantation studies of microglia into larval or adult brains followed by gene expression analysis might be a way to address this question in more detail.

In order to understand the degree of conservation between larval zebrafish microglia and mammalian microglia, we compared the gene expression profile of larval zebrafish microglia with previously published gene expression data sets from mouse and human microglia. Importantly, the global comparison of the gene expression in larval zebrafish microglia to the gene expression in mouse and human microglia showed a strong correlation. Furthermore, we find a significant enrichment of genes that are shared between larval zebrafish microglia and adult mouse or human microglia. This analysis revealed that the microglia core signature is conserved between larval zebrafish microglia and mouse and human microglia. Furthermore, GO category analysis showed that the shared genes are mainly representing “cellular process,” “development,” “metabolic process,” and “immune system process,” highlighting the importance of these processes in defining the identity of microglial cells. Further analysis revealed a number of core genes within these processes that were shared between the species, suggesting a conserved role for these genes in microglia biology. Recent single‐cell RNA sequencing studies of rodent and human microglia revealed specific time‐ and region‐dependent subtypes of microglia (Hammond et al., 2019; Jordão et al., 2019; Li et al., 2019; Masuda et al., 2019). Future studies will have to reveal if similar subtypes also exist during zebrafish development.

It should be noted that the comparison of microglia gene expression profiles among different species as presented here has its limitations. The different data sets were produced using slightly different methods to isolate microglia, perform RNA sequencing, and data analysis. This might impact on the number of differentially expressed genes identified in microglia and should be taken into consideration.

In conclusion, our results show that larval zebrafish microglia mature rapidly and express the core microglia signature, which is conserved across species. The combination of our newly acquired gene expression data and existing transgenic lines to specifically label microglia further strengthens the larval zebrafish model. Similarities in gene expression between larval zebrafish microglia and mammalian microglia will help to explore the function of these genes in more detail. The recent advances in CRISPR technology combined with high‐resolution live imaging facilitate mechanistic in vivo studies in the larval zebrafish model. This will not only allow studying microglia in physiology but also in the various disease contexts.

## Supporting information


**Figure S1** FACS gating strategy for microglia sorting from 5 dpf zebrafish larvae. (a) Brain cells were first gated to exclude debris, doublets (FSC Singlet; SSC Singlet) and dead cells (DAPI+). Unstained sample and cells incubated with secondary antibody only were used as controls to draw the gate corresponding to microglia. The same gating strategy has been used to isolate microglia from 3 and 7 dpf zebrafish larvae. (b) Median of fluorescence intensity of 3, 5, and 7 dpf microglia (Alx647+) was measured and does not show statistically significant difference between those three time points. The means ± SD of three independent experiments are plotted

**Figure S2** Purity of isolated microglia from 7 dpf zebrafish larvae. (a) Representative confocal images of Tg(XIa.Tubb:dsRED) and *Et(Zic4:Gal4TA4*,*UAS:mCherry)* ^*hmz5*^ larvae are shown to illustrate fluorescence from neurons (DsRed+) and radial glial cell progenitors (mCherry+) within the brain respectively. Scale bar represents 50 μm. (b‐i) The analysis of microglia and neuron populations of 7 dpf Tg(XIa.Tubb:dsRED) larvae reveals that a small population of DsRed+ cells (1.1%) appears positive for the microglial 4C4 antigen (Alx647+). This corresponds to 33 cells in the shown experiment. (b‐ii) These cells were isolated then analyzed by confocal microscopy. Their projection view and 3D reconstitution revealed that the DsRed signal corresponds to phagocytosed neurons by microglia (Alx647+). Scale bar represents 1 μm. (c‐i) The analysis of microglia and radial glial cell progenitor populations of 7 dpf *Et(Zic4:Gal4TA4*,*UAS:mCherry)* ^*hmz5*^ larvae reveals that a small population of mCherry+ cells (2.89%) appears positive for the microglial 4C4 antigen (Alx647+). This corresponds to 42 cells in the shown experiment. (c‐ii) These cells were isolated then analyzed by confocal microscopy. Their projection view and 3D reconstitution revealed that the mCherry signal corresponds to phagocytosed radial glial cell progenitors by microglia (Alx647+). Scale bar represents 1 μm

**Figure S3** Correlation between biological replicates of the zebrafish microglia transcriptome at 3, 5, and 7 dpf, related to Figure 2. (a‐i) Normalized counts from 3 dpf replicates 1 and 2 and 1 and 3. (a‐ii) Normalized counts from 5 dpf replicates 1 and 2 and 1 and 3. (a‐iii) Normalized counts from 7 dpf replicates 1 and 2 and 1 and 3. Pearson's *r* > .8 is indicated. Colors represent point density (Dark blue: low; light blue: high).

**Figure S4**: FACS gating strategy for macrophages isolated from 28 hpf zebrafish larvae. (a) Full embryo cells were first gated to exclude debris, doublets (FSC Singlet; SSC Singlet), and dead cells (DAPI+). Unstained sample was used as control to draw the gate to isolate macrophages (eGFP+)

**Figure S5** Zebrafish microglia transcriptome at 3, 5, and 7 dpf versus other brain cells from adult zebrafish. (a) Principal component analysis (PCA) score plot obtained from normalized counts of isolated microglia from 600 zebrafish embryos at 3 (green), 5 (yellow), and 7 (magenta) dpf (*N* = 3) and normalized counts of isolated other brain cells [blue] from Oosterhof et al., (2016). The PCA score plot shows that samples from isolated microglia RNA are grouped together compared to sample of RNA from other brain cells. (b) Venn diagram showing unique and intersecting genes from microglia transcriptome at 3, 5, and 7 dpf and other brain cells. No significant gene enrichments (FDR < 0.05) were observed

**Table S1**

**Table S2**

**Table S3**

**Table S4**

**Table S5**

**Table S6**

**Table S7**

**Table S8**

**Table S9**

**Table S10**

**Table S11**

**Table S12**

## Data Availability

The data that support the findings of this study are included in the supplementary tables and are available from the corresponding author upon reasonable request.
