# Supplementary figures and images for: Gene expression profiling reveals a conserved microglia signature in larval zebrafish
Source: Glia. 2019 Sep 11;68(2):298–315. doi: 10.1002/glia.23717 (PMC6916425; doi:10.1002/glia.23717)

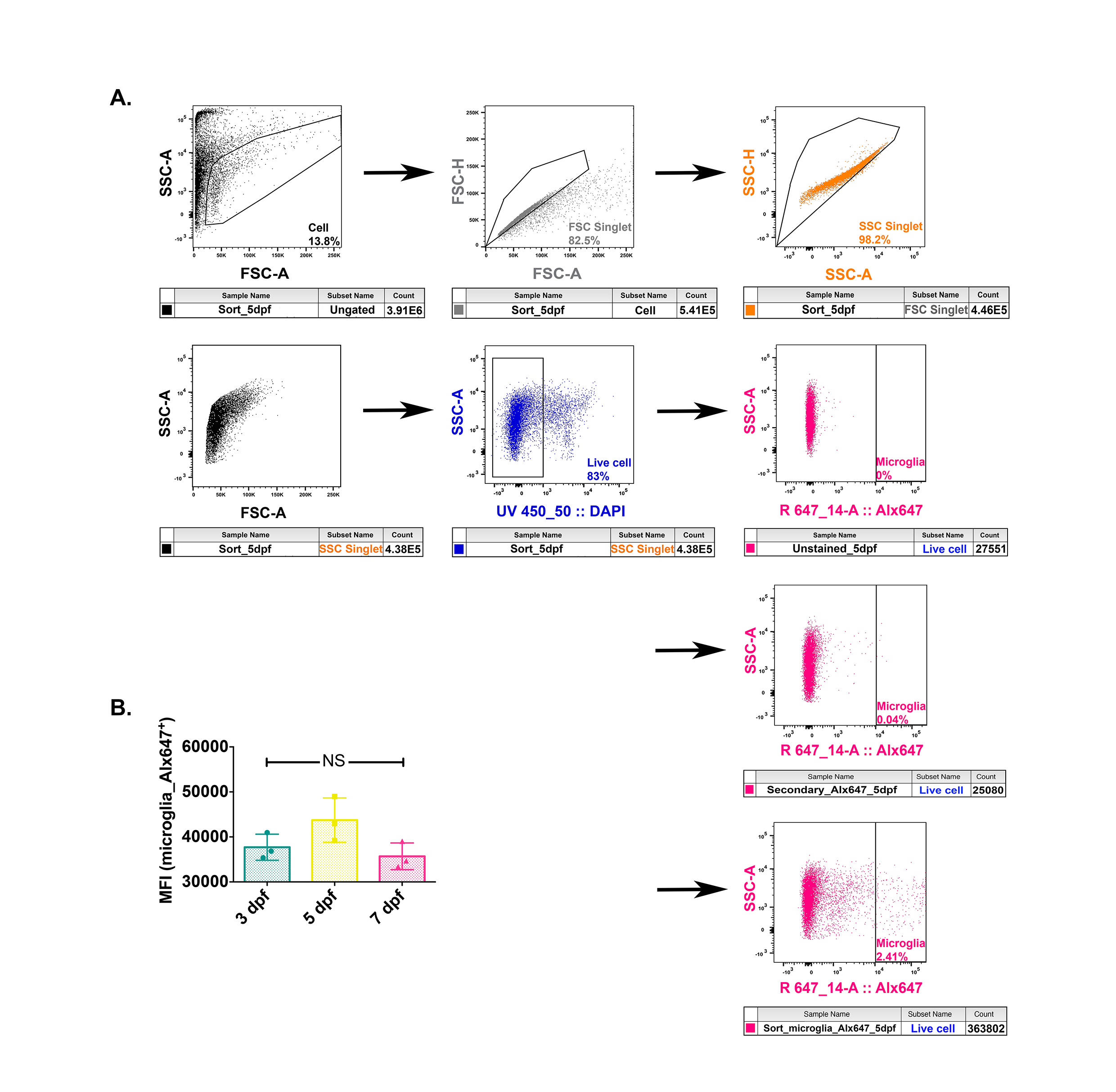

Supplement: Supplementary file 1 — Figure S1 FACS gating strategy for microglia sorting from 5 dpf zebrafish larvae. (a) Brain cells were first gated to exclude debris, doublets (FSC Singlet; SSC Singlet) and dead cells (DAPI+). Unstained sample and cells incubated with secondary antibody only were used as controls to draw the gate corresponding to microglia. The same gating strategy has been used to isolate microglia from 3 and 7 dpf zebrafish larvae. (b) Median of fluorescence intensity of 3, 5, and 7 dpf microglia (Alx647+) was measured and does not show statistically significant difference between those three time points. The means ± SD of three independent experiments are plotted [file GLIA-68-298-s001.tif]

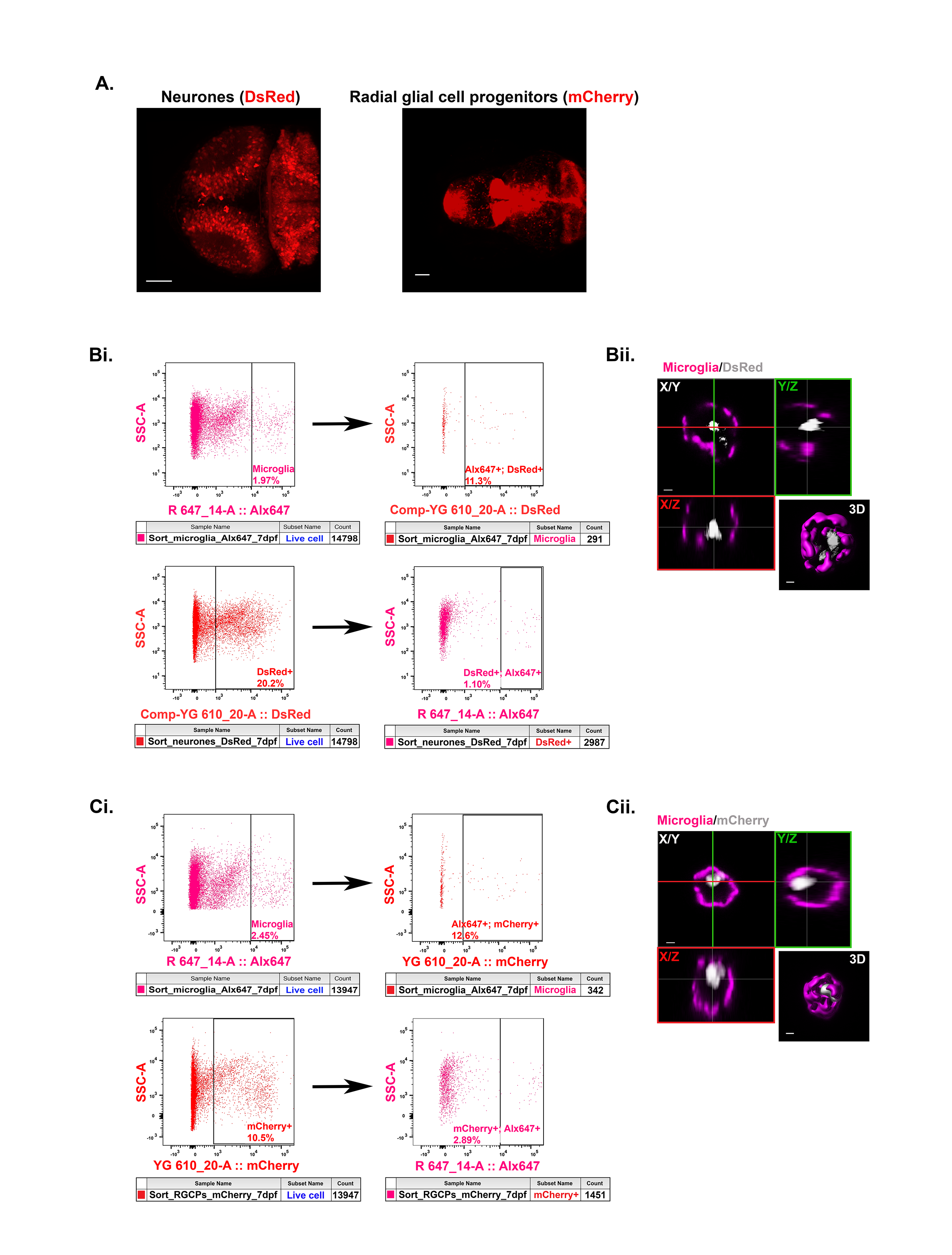

Supplement: Supplementary file 2 — Figure S2 Purity of isolated microglia from 7 dpf zebrafish larvae. (a) Representative confocal images of Tg(XIa.Tubb:dsRED) and Et(Zic4:Gal4TA4,UAS:mCherry) hmz5 larvae are shown to illustrate fluorescence from neurons (DsRed+) and radial glial cell progenitors (mCherry+) within the brain respectively. Scale bar represents 50 μm. (b‐i) The analysis of microglia and neuron populations of 7 dpf Tg(XIa.Tubb:dsRED) larvae reveals that a small population of DsRed+ cells (1.1%) appears positive for the microglial 4C4 antigen (Alx647+). This corresponds to 33 cells in the shown experiment. (b‐ii) These cells were isolated then analyzed by confocal microscopy. Their projection view and 3D reconstitution revealed that the DsRed signal corresponds to phagocytosed neurons by microglia (Alx647+). Scale bar represents 1 μm. (c‐i) The analysis of microglia and radial glial cell progenitor populations of 7 dpf Et(Zic4:Gal4TA4,UAS:mCherry) hmz5 larvae reveals that a small population of mCherry+ cells (2.89%) appears positive for the microglial 4C4 antigen (Alx647+). This corresponds to 42 cells in the shown experiment. (c‐ii) These cells were isolated then analyzed by confocal microscopy. Their projection view and 3D reconstitution revealed that the mCherry signal corresponds to phagocytosed radial glial cell progenitors by microglia (Alx647+). Scale bar represents 1 μm [file GLIA-68-298-s002.tif]

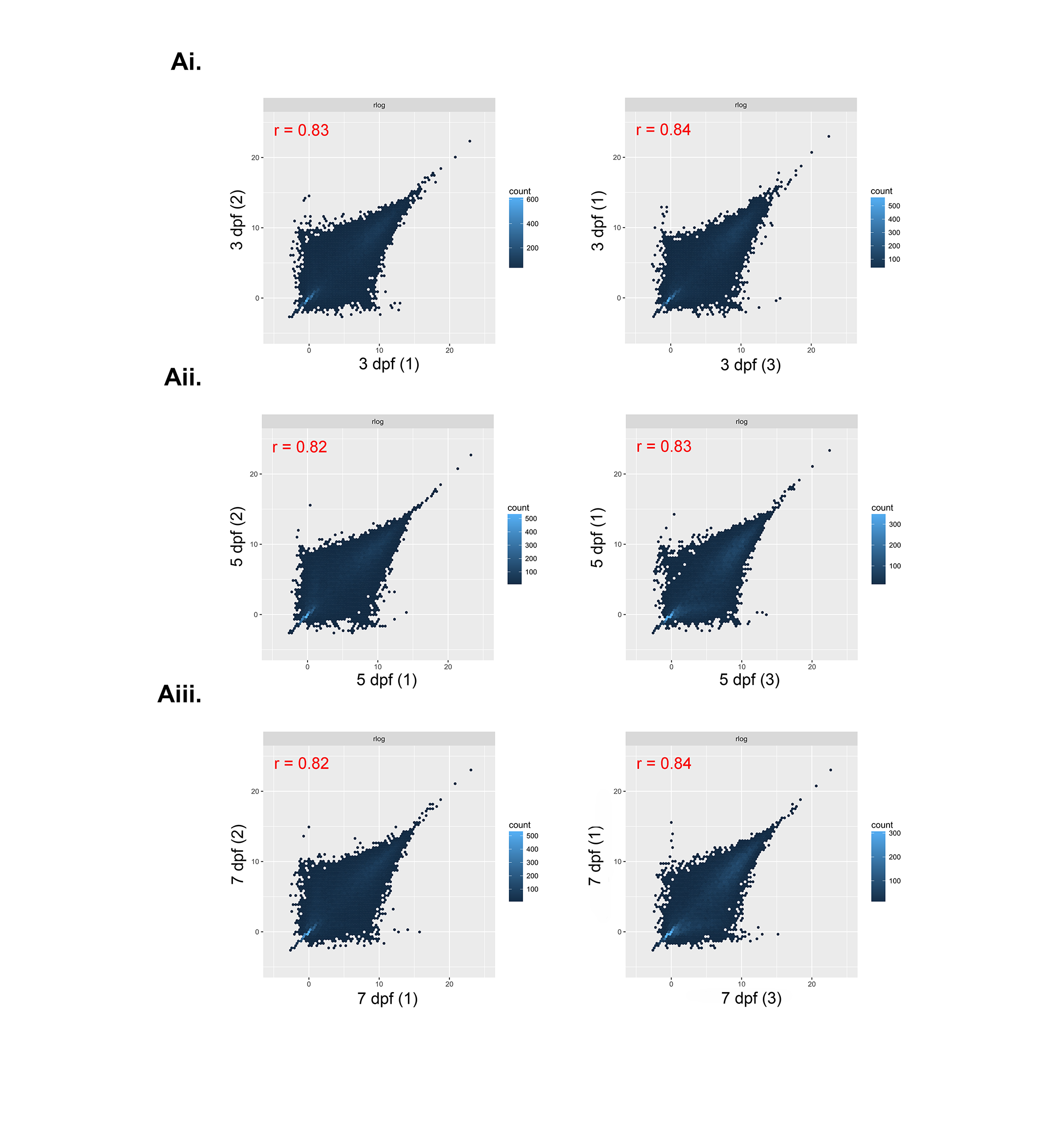

Supplement: Supplementary file 3 — Figure S3 Correlation between biological replicates of the zebrafish microglia transcriptome at 3, 5, and 7 dpf, related to Figure 2. (a‐i) Normalized counts from 3 dpf replicates 1 and 2 and 1 and 3. (a‐ii) Normalized counts from 5 dpf replicates 1 and 2 and 1 and 3. (a‐iii) Normalized counts from 7 dpf replicates 1 and 2 and 1 and 3. Pearson's r > .8 is indicated. Colors represent point density (Dark blue: low; light blue: high). [file GLIA-68-298-s003.tif]

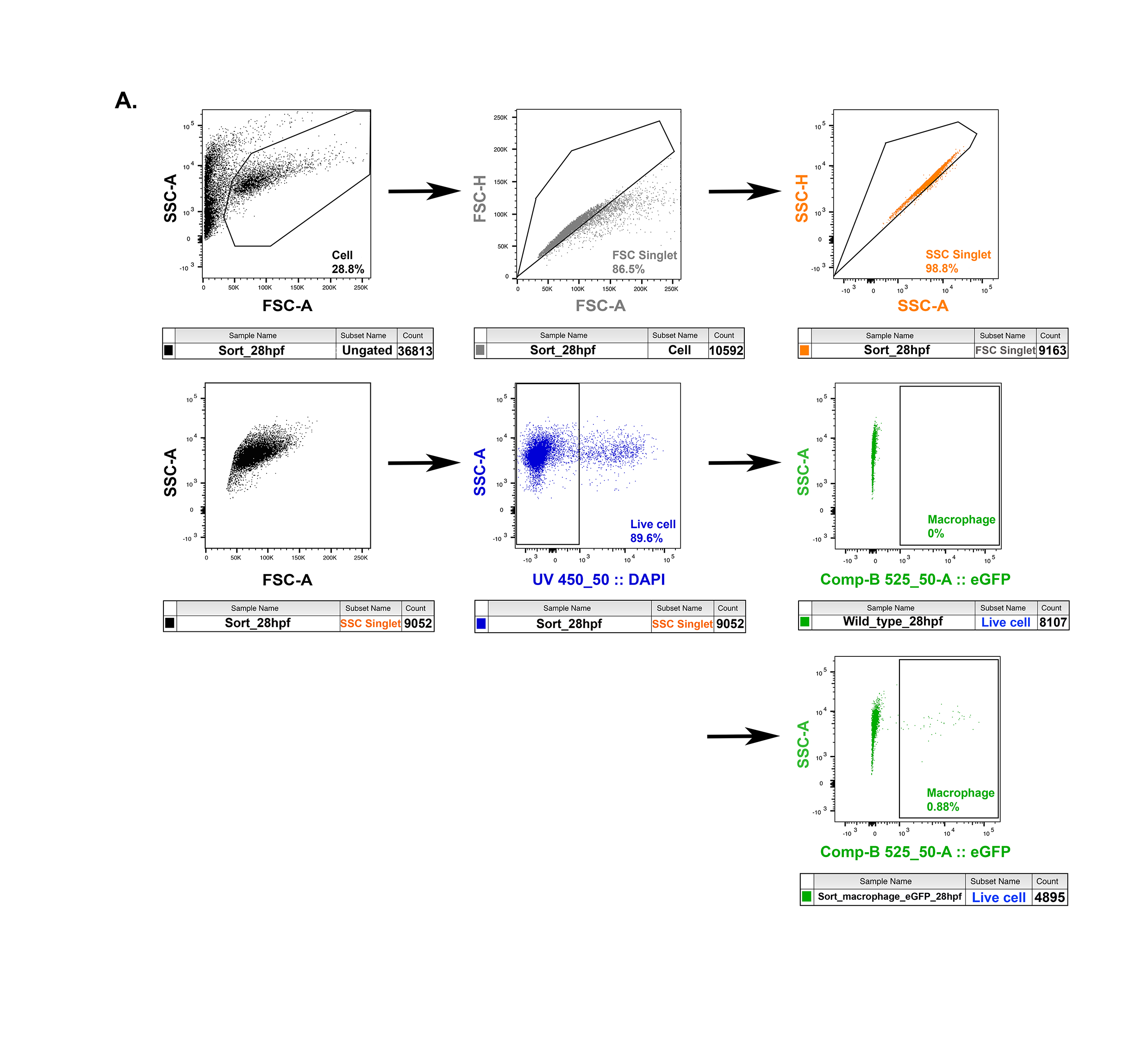

Supplement: Supplementary file 4 — Figure S4: FACS gating strategy for macrophages isolated from 28 hpf zebrafish larvae. (a) Full embryo cells were first gated to exclude debris, doublets (FSC Singlet; SSC Singlet), and dead cells (DAPI+). Unstained sample was used as control to draw the gate to isolate macrophages (eGFP+) [file GLIA-68-298-s004.tif]

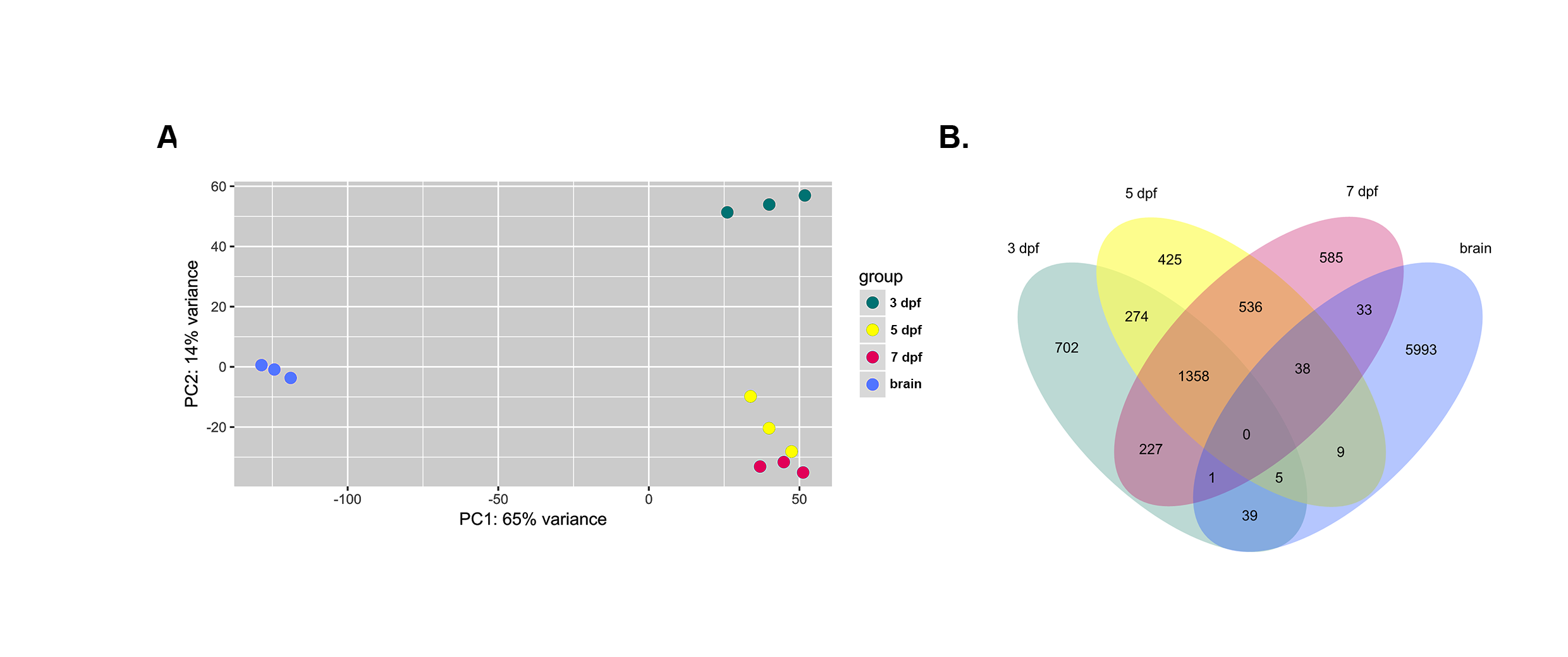

Supplement: Supplementary file 5 — Figure S5 Zebrafish microglia transcriptome at 3, 5, and 7 dpf versus other brain cells from adult zebrafish. (a) Principal component analysis (PCA) score plot obtained from normalized counts of isolated microglia from 600 zebrafish embryos at 3 (green), 5 (yellow), and 7 (magenta) dpf (N = 3) and normalized counts of isolated other brain cells [blue] from Oosterhof et al., (2016). The PCA score plot shows that samples from isolated microglia RNA are grouped together compared to sample of RNA from other brain cells. (b) Venn diagram showing unique and intersecting genes from microglia transcriptome at 3, 5, and 7 dpf and other brain cells. No significant gene enrichments (FDR < 0.05) were observed [file GLIA-68-298-s005.tif]
